# Dynamic regulation of hepatic lipid metabolism by torsinA and its activators

**DOI:** 10.1172/jci.insight.175328

**Published:** 2024-01-09

**Authors:** Antonio Hernandez-Ono, Yi Peng Zhao, John W. Murray, Cecilia Östlund, Michael J. Lee, Angsi Shi, William T. Dauer, Howard J. Worman, Henry N. Ginsberg, Ji-Yeon Shin

**Affiliations:** 1 Department of Medicine,; 2 Columbia Center for Human Development, and; 3 Department of Pathology and Cell Biology, Vagelos College of Physicians and Surgeons, Columbia University, New York, New York, USA.; 4 Department of Biostatistics, Mailman School of Public Health, Columbia University, New York, New York, USA.; 5 Peter O’Donnell Jr. Brain Institute,; 6 Department of Neurology, and; 7 Department of Neuroscience, University of Texas Southwestern Medical Center, Dallas, Texas, USA.

**Keywords:** Hepatology, Metabolism, Lipoproteins

## Abstract

Depletion of torsinA from hepatocytes leads to reduced liver triglyceride secretion and marked hepatic steatosis. TorsinA is an atypical ATPase that lacks intrinsic activity unless it is bound to its activator, lamina-associated polypeptide 1 (LAP1) or luminal domain–like LAP1 (LULL1). We previously demonstrated that depletion of LAP1 from hepatocytes has more modest effects on liver triglyceride secretion and steatosis development than depletion of torsinA. We now show that depletion of LULL1 alone does not significantly decrease triglyceride secretion or cause steatosis. However, simultaneous depletion of both LAP1 and LULL1 leads to defective triglyceride secretion and marked steatosis similar to that observed with depletion of torsinA. Depletion of both LAP1 and torsinA from hepatocytes generated phenotypes similar to those observed with only torsinA depletion, implying that the 2 proteins act in the same pathway in liver lipid metabolism. Our results demonstrate that torsinA and its activators dynamically regulate hepatic lipid metabolism.

## Abstract 2

Loss of torsinA or both of its activators, LAP1 and LULL1, promotes steatosis via lateration in hepatic lipid secretion.

## Introduction

Abnormal hepatic lipid metabolism leads to steatotic liver disease (SLD) that can progress to steatohepatitis and cirrhosis (1–3). SLD is a complex systemic disease, with many extrahepatic and intrahepatic factors contributing to the accumulation of lipids in hepatocytes (4, 5). While some of the diverse factors that affect liver lipid metabolism have been identified, the precise cell biological mechanisms that lead to excess accumulation of fat in the liver are incompletely understood. We previously demonstrated that a nuclear envelope–localized protein complex of torsinA and one of its activators, lamina-associated polypeptide 1 (LAP1), is an important hepatocyte-intrinsic factor in the regulation of intrahepatic triglyceride (TG) metabolism (6).

TorsinA is an ATPase associated with various cellular activities (AAA+) that resides in the endoplasmic reticulum (ER) lumen and continuous perinuclear space of the nuclear envelope (7–9). TorsinA interacts with at least 2 differentially localized transmembrane proteins that contain highly conserved luminal domains. In the perinuclear space, it interacts with LAP1 at the inner nuclear membrane and with luminal domain–like LAP1 (LULL1) throughout the entire ER (10). In vitro, torsinA does not display ATPase activity in isolation; however, ATP hydrolysis is induced upon association with the luminal domains of either LAP1 or LULL1 (11, 12). Structural biology studies have shown that torsinA combines with these transmembrane proteins to form heterohexameric rings in which LAP1 and LULL1 induce ATPase activity by supplying an arginine to the neighboring torsinA molecule (12). A 3–base pair deletion (ΔGAG) in *TOR1A* encoding torsinA, resulting in loss of a glutamic acid at residues 302/303 in the carboxyl terminus of torsinA, causes the autosomal dominant movement disorder early-onset DYT1 dystonia (13). Data from genetically modified mice demonstrate that this variant leads to loss of torsinA function in maintaining neuronal nuclear envelope morphology (14). The role of LAP1 and LULL1 in activating torsinA is further supported by the finding that deletion of glutamic acids 302/303 from torsinA impairs its ATP-dependent interaction with these proteins (15, 16). However, even with the knowledge provided by these elegant biochemical, structural biological, and cell biology studies, the in vivo functional relevance of the interaction between torsinA and its activators is largely unknown.

We have identified what we believe are novel roles for torsinA and LAP1 in liver lipid metabolism. Mice with hepatocyte-specific depletion of torsinA (A-CKO mice) have markedly reduced secretion of TG-rich VLDL and severe steatosis, while livers from mice with hepatocyte-specific LAP1 deletion (L-CKO mice) demonstrate modest reductions in VLDL secretion and less severe steatosis (6). We took advantage of these mammalian systems with measurable readouts to investigate the unresolved functional significance of the torsinA activators LAP1 and LULL1 in vivo. Given the more modest decrease in VLDL secretion and less severe steatosis in livers of L-CKO mice compared with A-CKO mice, we reasoned that LULL1 may provide some degree of torsinA activation in the absence of LAP1. We therefore hypothesized that the combined depletion of LAP1 and LULL1 from hepatocytes is sufficient to abolish all torsinA function and phenocopy A-CKO mice that have depletion of torsinA from hepatocytes. We also hypothesized that combined depletion of LAP1 and torsinA would lead to the same phenotypes observed with depletion of only torsinA from hepatocytes. We have now tested these hypotheses in vivo.

## Results

### Mice with depletion of LULL1 from hepatocytes have no steatosis and normal VLDL secretion.

**Figure 1:**
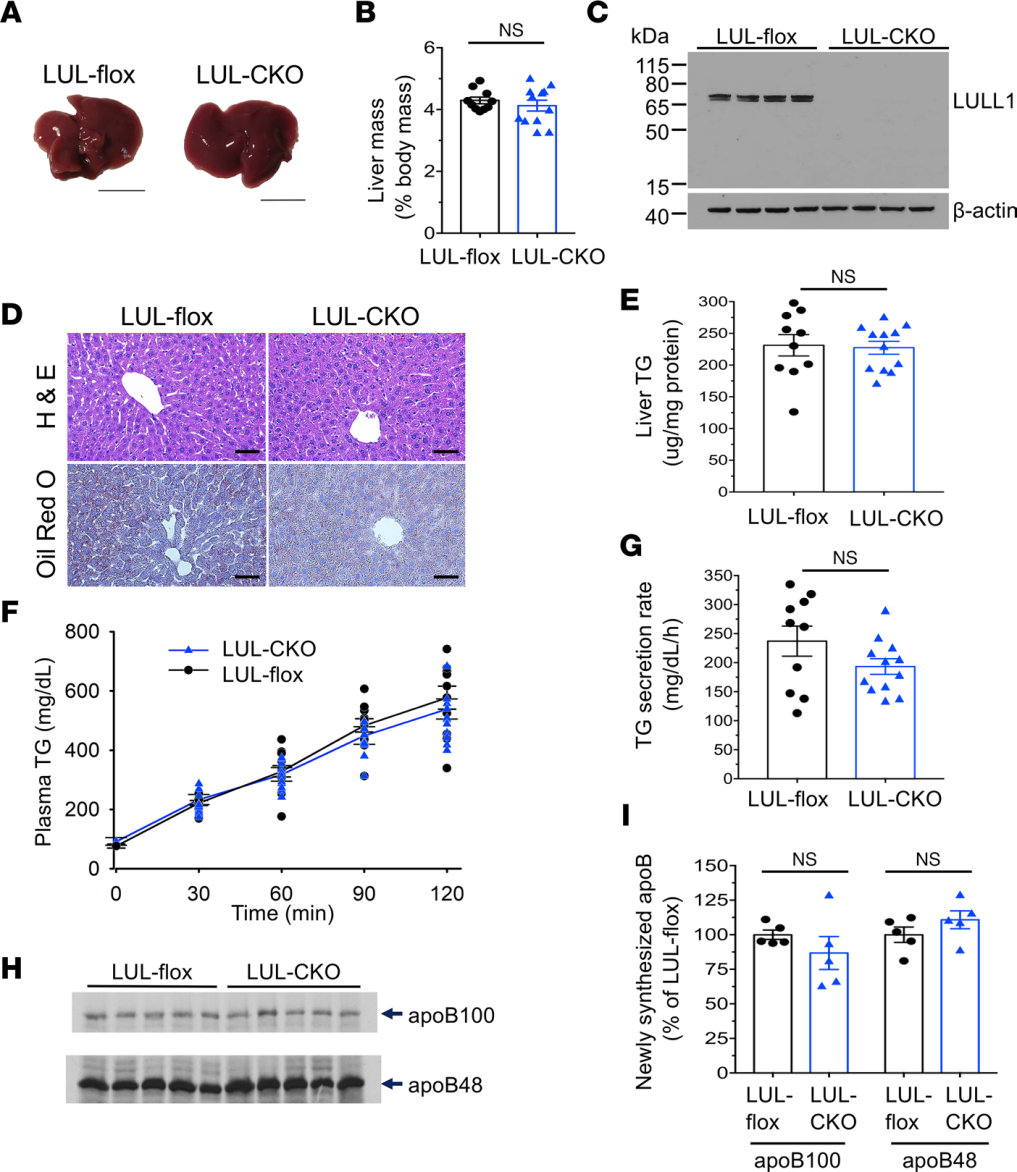
Mice with depletion of LULL1 from hepatocytes show no overt liver abnormalities. (**A**) Representative photographs of livers from 4-month-old control (LUL-flox) and LUL-CKO mice fed a chow diet. Scale bars: 1 cm. (**B**) Liver-to-body mass ratios of LUL-flox (*n* = 10) and LUL-CKO mice (*n* = 12). (**C**) IB of liver lysates from LUL-flox and LUL-CKO mice probed with Abs against LULL1 and β-actin. Each lane is a sample from a different mouse. The anti-LULL1 Ab detects 2 closely migrating bands (14). (**D**) Representative light photomicrographs of liver sections from chow-fed mice stained with H&E or Oil Red O. Scale bars: 50 μm. (**E**) Liver TG content of LUL-flox (*n* = 10) and LUL-CKO (*n* = 12) mice. Mice were fasted for 4–5 hours before collecting livers to measure TG content. (**F**) Plasma TG concentration versus time after injection of tyloxapol to block peripheral uptake in LUL-CKO (*n* = 12) and LUL-flox (*n* = 10) mice. (**G**) TG secretion rates calculated from the changes in plasma concentrations between 30 and 120 minutes in **F**. (**H**) Autoradiogram of SDS-polyacrylamide gel showing ^35^S-labeled plasma proteins collected 120 minutes after injection with ^35^S-methionine and tyloxapol. Each lane shows proteins from an individual mouse. Migrations of ^35^S-methionine–labeled apoB100 and apoB48 are indicated (*n* = 5 mice per group). (**I**) Bands corresponding to apoB100 and apoB48 shown in panel **F** were quantified by densitometry and shown as a percentage of the value from the LUL-flox group (*n* = 5 mice per group). In panels **B**, **E**–**G**, and **I**, values are mean ± SEM, with each circle or triangle representing the value from an individual mouse. NS, not significant by unpaired, 2-tailed Student’s *t* test.

A-CKO mice with hepatocyte-specific depletion of torsinA develop striking hepatic steatosis, while L-CKO mice with depletion of LAP1 have a relatively mild steatosis phenotype (6). This suggests that LULL1 may activate torsinA in the ER or continuous nuclear envelope, providing partial cellular activity. To investigate the potential role of LULL1 in hepatic steatosis and lipoprotein secretion, we crossed mice with 2 floxed alleles of *Tor1aip2* encoding LULL1 (LUL-flox mice) with mice with an albumin-Cre transgene to obtain mice with depletion of LULL1 from hepatocytes (LUL-CKO mice) (Supplemental Figure 1 and Supplemental Table 1; supplemental material available online with this article; https://doi.org/10.1172/jci.insight.175328DS1). Both male and female LUL-CKO mice developed normally, without overtly abnormal phenotypes. Given the absence of apparent sex-specific differences, we subsequently analyzed them in an aggregated manner. Livers from LUL-CKO mice at 4 months of age were grossly normal (Figure 1A) and mean liver-to-body mass ratio was the same as littermate controls (Figure 1B). Immunoblotting showed that LULL1 expression was detectable only at very low levels in livers from LUL-CKO mice (Figure 1C), with only a weak signal present in overexposed immunoblots (IBs) (Supplemental Figure 2A), likely resulting from expression in liver cell types other than hepatocytes. In control mice, our anti-LULL1 Ab detected 2 closely migrating bands, as shown in a previous report (14). On histological examination, liver sections from LUL-CKO mice stained with H&E or Oil Red O to detect fat did not appear different from those from littermate controls (Figure 1D). Based on interpretation of the sections stained with H&E, a pathologist blinded to genotype scored liver steatosis (on a scale of 0 to 3) as 0.5 ± 0.14 in the LUL-flox mice and 0.75 ± 0.13 in LUL-CKO mice (mean ± SEM, *n* = 4, *P* value not significant by Student’s *t* test). The steady-state liver TG content in LUL-CKO mice was also similar to that in control mice (Figure 1E). To assess liver VLDL secretion, we measured plasma TG concentrations following intravenous administration of tyloxapol to block both lipolysis of VLDL TG and uptake of circulating TG-rich VLDL remnant lipoproteins. There were no differences in plasma TG concentration between LUL-CKO and controls at all time points measured after tyloxapol administration (Figure 1F). The calculated hepatic TG secretion rate was the same in LUL-CKO and control mice (Figure 1G). Analysis of plasma proteins by SDS-PAGE followed by autoradiography 120 minutes after injection of ^35^S-methionine with tyloxapol showed no significant differences in radiolabeled apolipoprotein B100 (apoB100) or apoB48 (Figure 1, H and I). ApoB100 and apoB48 are major protein components of mouse VLDL (17, 18). These results show that depletion of LULL1 from hepatocytes does not lead to steatosis or decreased VLDL secretion. There were no differences in plasma glucose and insulin concentrations between LUL-CKO and control mice (Supplemental Figure 3).

### Hepatocytes from LUL-CKO mice do not have increased TG accumulation and have normal torsinA and LAP1 distribution.

**Figure 2:**
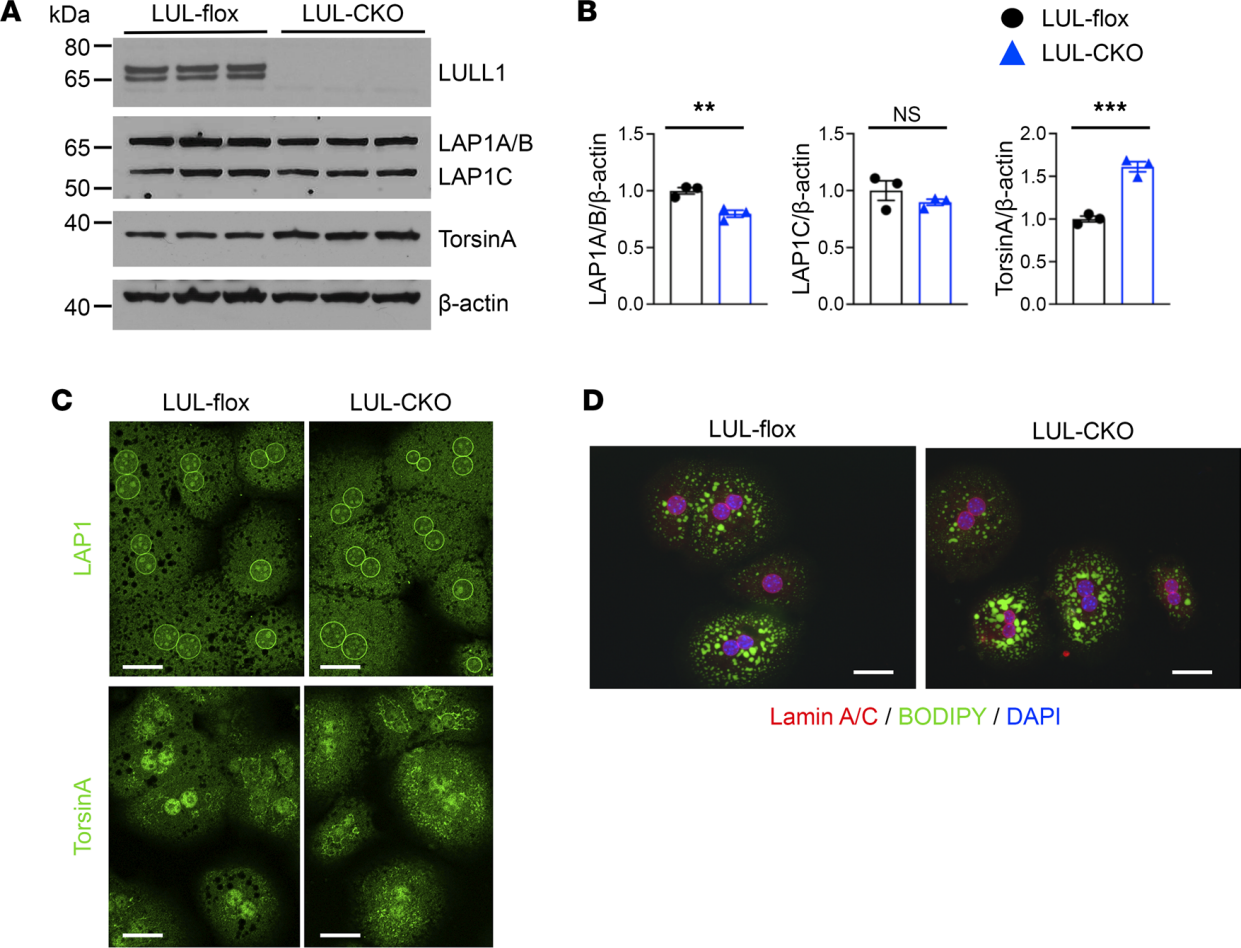
Expression of torsinA and LAP1 in livers and hepatocytes of LUL-CKO mice. (**A**) IBs of cell lysates from hepatocyte cultures from LUL-flox and LUL-CKO mice at 4 months of age. Blots were probed with Abs against LULL1, LAP1, torsinA, and β-actin. (**B**) Relative ratios of band densities of LAP1A/B, LAP1C, or torsinA versus β-actin blots shown in panel **A** (*n* = 3 different hepatocyte cultures from 1 mouse in each group). Values are mean ± SEM, with individual circles or triangles representing the value from each lane on the IBs in panel **A**. **P < 0.01, ***P < 0.001 by unpaired, 2-tailed Student’s *t* test. NS, not significant. (**C**) Confocal immunofluorescence micrographs of isolated hepatocytes labeled with anti-LAP1 (green, upper panels) and anti-torsinA (green, bottom panels) Abs. Scale bars: 20 μm. (**D**) Confocal immunofluorescence micrographs of hepatocytes stained with anti–lamin A/C Ab (red), BODIPY (green), and DAPI (blue). Scale bars: 20 μm.

LULL1 overexpression promotes torsinA redistribution to the nuclear envelope in cultured cells (16, 19). Therefore, we examined whether the expression or distribution of endogenous torsinA or LAP1 is changed in LULL1-deficient hepatocytes. Immunoblotting of whole-liver lysates from LUL-CKO mice showed depletion of LULL1, no changes in LAP1, and a slight but significant increase in torsinA expression (Supplemental Figure 4, A and B). Immunoblotting of lysates of primary hepatocytes isolated from LUL-CKO mice showed depletion of LULL1, a slight decrease in LAP1, and a slight but significant increase in total torsinA expression (Figure 2, A and B). Immunofluorescence microscopy did not identify obvious changes in the distribution of endogenous LAP1 or torsinA in hepatocytes isolated from LUL-CKO mice (Figure 2C). While the distribution of the torsinA immunofluorescence signal was not changed in hepatocytes from LUL-CKO mice, its cytoplasmic mean intensity was increased in LULL1-depleted hepatocytes, consistent with immunoblotting results (Supplemental Figure 4, C and D). Depletion of LAP1, another activator in the nuclear envelope, also did not lead to obvious changes in the distribution or intensity of torsinA in the ER of hepatocytes (Supplemental Figure 4, E and F). Hence, endogenous torsinA distribution in the ER was not dramatically changed in hepatocytes with depletion of either of its activators. BODIPY staining of neutral lipids in hepatocytes isolated from LUL-CKO mice did not show nuclear lipid droplets, which are observed in mice with hepatocyte-specific depletion of LAP1 (6, 20), or an increase in cytosolic lipid content compared with littermate controls (Figure 2D). We quantified BODIPY fluorescence intensity using an automated measuring method (Supplemental Figure 5A). The results did not show a difference in mean fluorescence intensities in LUL-flox and LUL-CKO mice (Supplemental Figure 5B). These results show that depletion of LULL1 from hepatocytes does not lead to excess lipid accumulation and that LAP1 may be sufficient to stimulate torsinA activity when LULL1 is depleted.

As hepatocytes isolated from LUL-CKO mice did not differ in lipid content compared to LUL-flox controls, we tested whether this was a result of lower expression of LULL1 compared with LAP1 in mouse liver. Although immunoblotting does not allow direct comparison of the expression level of 2 proteins, we detected robust expression of LULL1 and LAP1 in WT mouse liver tissue and hepatocyte lysates. Further examination of mRNA expression showed similar levels of those encoding LAP1B and LAP1C, 2 LAP1 isoforms, and LULL1 in livers from WT mice at 4 months of age (Supplemental Table 2 and Supplemental Table 3). Mouse ENCODE transcriptome data also show similar expression of the 2 genes in adult liver (*Lap1b* RPKM 5.7, *Lull1* RPKM 6.5) (21). These data indicate that the minimal effects of LULL1 depletion on lipid secretion and accumulation are not likely explained by large differences in protein expression levels. The absence of excess lipid accumulation in hepatocytes lacking LULL1 suggests that LAP1 may sufficiently stimulate torsinA activity in the absence of LULL1. However, the more modest steatosis observed in mice with hepatocyte-specific depletion of LAP1 compared with those with depletion of torsinA suggests that LULL1 alone nonetheless provides partial torsinA function when present in cells.

We next tested whether LAP1 or LULL1 depletion affects the interaction of the other protein with torsinA in hepatocytes. We performed co-IP with anti-FLAG Ab after adenoviral vector–mediated expression of FLAG-torsinA in control, LAP1-depleted, and LULL1-depleted hepatocytes. Endogenous LAP1 in both control and LULL1-deleted hepatocytes coprecipitated with FLAG-torsinA, as did endogenous LULL1 in control and L-CKO hepatocytes with depletion of LAP1 (Supplemental Figure 6).

### Depletion of both LAP1 and LULL1 from mouse hepatocytes leads to steatosis similar to that occurring with torsinA depletion.

We hypothesized that if LAP1 and LULL1 are the only or major activators of torsinA in hepatocytes, then depletion of both will phenocopy the marked hepatic steatosis and VLDL secretion defect in A-CKO mice with depletion of torsinA from hepatocytes. To generate combined depletion of both proteins, we used shRNA-mediated gene depletion, as we could not delete the genes encoding both LAP1 and LULL1 by crossing mouse lines due to their tight physical linkage in the genome (10). We designed 4 shRNAs to deplete LAP1 and selected one with the highest knockdown efficiency in a mouse cell line (Supplemental Figure 7A). We then generated an adenoviral vector to express the shRNA under the control of a U6 promoter (Ad-Lap1) for in vivo depletion of LAP1 from mouse hepatocytes. To test the efficiency of Ad-Lap1, we transduced cultured WT primary hepatocytes with this or a similar construct expressing a control scrambled shRNA (Ad-ctrl) and confirmed the depletion of LAP1 48 hours later by immunoblotting (Supplemental Figure 7, B and C). Accordingly, immunofluorescence microscopy studies showed no nuclear rim staining of LAP1 in the hepatocytes transduced with Ad-Lap1 (Supplemental Figure 7D). We then intravenously administrated the adenoviral constructs to WT mice to test in vivo efficacy of LAP1 depletion. Analysis of liver sections by immunofluorescence microscopy 5 days after injection demonstrated depletion of LAP1 (Supplemental Figure 7E).

**Figure 3:**
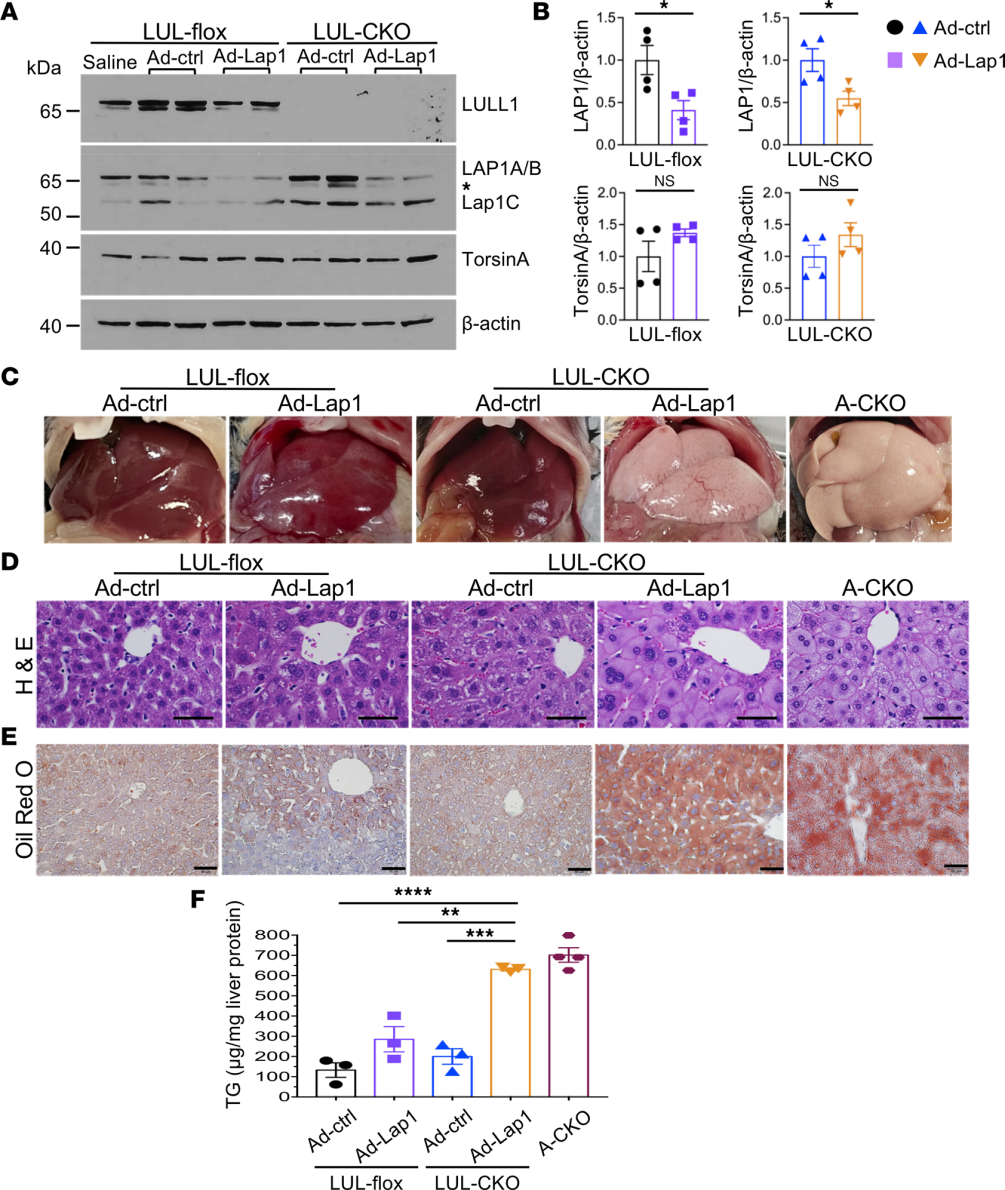
Depletion of LAP1 and LULL1 from hepatocytes leads to liver steatosis similar to that occurring with torsinA depletion. (**A**) IB of liver lysates from LUL-flox and LUL-CKO mice administered adenoviral vectors expressing either control scrambled shRNA (Ad-ctrl) or shRNA that targets LAP1 (Ad-Lap1). Lysates from liver of a LULL-flox mouse treated with saline is also included as a control. Abs against LULL1, LAP1, torsinA, and β-actin were used. *Indicates nonspecific bands often detected in whole-liver lysates using polyclonal Abs against LAP1. (**B**) Relative ratios of band densities of LAP1 (all isoforms) or torsinA versus β-actin in blots as shown in panel **A**. Band densities were measured from 2 independent IBs using protein extracts from 2 mice per group each. Values are mean ± SEM, with each circle, square, or triangle representing the value from each IB (*n* = 4). *P < 0.05 by unpaired, 2-tailed Student’s *t* test. NS, not significant. (**C**) Representative photographs of livers from LUL-flox and LUL-CKO mice injected with Ad-ctrl or Ad-Lap1. Photograph of a liver from an A-CKO mouse with depletion of torsinA from hepatocytes is shown for comparison. (**D**) Representative light photomicrographs of liver sections stained with H&E from LUL-flox and LUL-CKO mice administered Ad-Lap1 or Ad-ctrl. Section from an A-CKO mouse is shown for comparison. Scale bars: 50 μm. (**E**) Representative light photomicrographs of liver sections stained with Oil Red O from LUL-flox and LUL-CKO mice injected with adenoviruses expressing either Ad-ctrl or Ad-Lap1. A section from an A-CKO mouse is shown for comparison. Scale bars: 50 μm. (**F**) TG content in livers from LUL-flox and LUL-CKO mice administered Ad-ctrl or Ad-Lap1. Mice were fasted for 4–5 hours before isolating livers. Liver TG values from 4 A-CKO mice were included for comparison. Values are mean ± SEM, with each symbol representing the value from an individual mouse (*n* = 3-4). **P < 0.01; ***P < 0.001; ****P < 0.0001 by 1-way ANOVA followed by Tukey’s post hoc test.

After confirming the efficacy of Ad-Lap1 in depleting LAP1, we administered it (or Ad-ctrl) intravenously to LUL-flox and LUL-CKO mice. Immunoblotting of whole-liver lysates showed that LUL-flox and LUL-CKO mice treated with Ad-Lap1 had reductions in expression in all LAP1 isoforms, with a somewhat greater decrease in LAP1C in the LUL-flox mice (Figure 3A). Quantification of total LAP1 signals on IBs of whole-liver lysates showed that Ad-Lap1 treatment produced approximately 50% reductions in LAP1 expression in LUL-flox and LUL-CKO mice, and a slight but nonsignificant trend toward increased torsinA expression in LUL-CKO mice (Figure 3B). On gross inspection 5 days after adenoviral vector treatment, livers from LUL-flox mice given Ad-ctrl or Ad-LAP1 and from LUL-CKO mice administered Ad-ctrl were grossly normal, whereas livers from LUL-CKO mice treated with Ad-Lap1, which have depletion of both LAP1 and LULL1 from hepatocytes, were grossly white, similar to livers from A-CKO mice with hepatocyte-specific depletion of torsinA (Figure 3C). Histological examination of sections stained with H&E showed increased fat content in livers from mice with depletion of both LAP1 and LULL1 from hepatocytes — similar to that in livers from A-CKO mice with depletion of torsinA — and a more modest increase in fat in mice with depletion of only LAP1 from hepatocytes (Figure 3D). A pathologist blinded to genotype and treatment invariably gave a steatosis score of 3 (on a scale of 0 to 3) to every section examined from livers of mice with combined depletion of LAP1 and LULL1 or with depletion of torsinA alone; the steatosis was mostly microvesicular. Micrographs of liver sections stained with Oil Red O confirmed significant fat in livers of mice with depletion of both LAP1 and LULL1 from hepatocytes, which was similar to those from A-CKO mice with depletion of torsinA (Figure 3E). Livers from mice with hepatocyte-specific depletion of both LAP1 and LULL1 had a similar level of TG compared to A-CKO mice, but significantly more TG compared with LUL-KO mice receiving Ad-ctrl or LUL-flox mice receiving either Ad-ctrl or Ad-LAP1 (Figure 3F). These results show that depletion of both known torsinA activators — LAP1 and LULL1 — from hepatocytes produces a dramatic degree of liver steatosis, similar to what occurs with depletion of torsinA.

**Figure 4:**
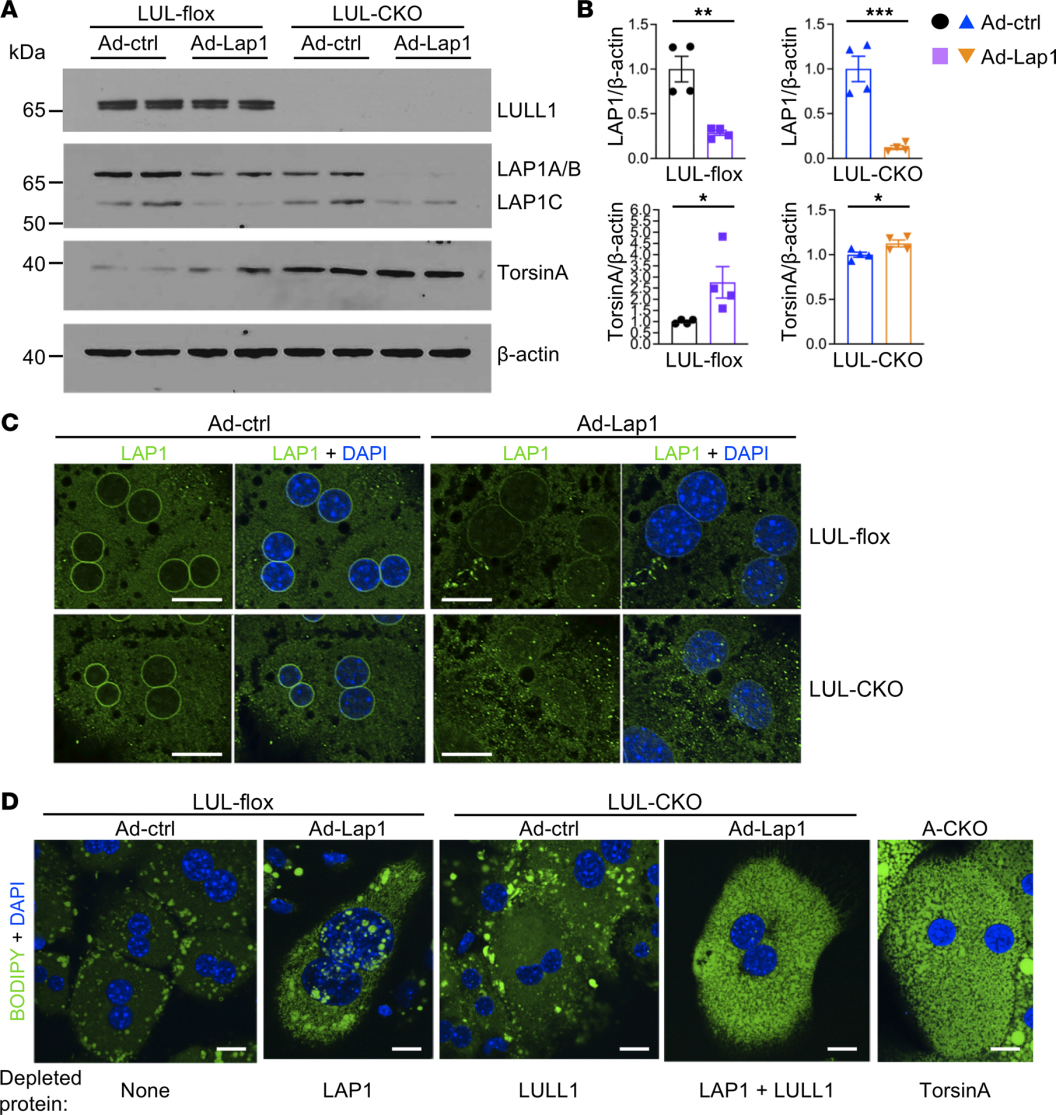
Depletion of LAP1 and LULL1 from hepatocytes leads to lipid droplet distribution similar to that occurring with torsinA depletion. (**A**) IBs of hepatocyte lysates from LUL-flox and LUL-CKO mice administered adenoviral vectors expressing either control scrambled shRNA (Ad-ctrl) or shRNA that targets LAP1 (Ad-Lap1). Abs against LULL1, LAP1, torsinA, and β-actin were used. (**B**) Relative ratios of band densities of LAP1 (all isoforms) or torsinA versus β-actin blots shown in panel **A**. Band densities were measured from 2 independent IBs using protein extracts from 2 mice per group each. Values are mean ± SEM, with each circle, square, or triangle representing the value from each IB (*n* = 4). *P < 0.05, **P < 0.01, ***P < 0.001 by unpaired, 2-tailed Student’s *t* test. (**C**) Representative immunofluorescence confocal photomicrographs of hepatocytes from LUL-flox and LUL-CKO mice administered Ad-ctrl or Ad-Lap1. Cells were stained with anti-LAP1 Ab (green) and DAPI (blue). Scale bars: 20 μm. (**D**) Representative immunofluorescence confocal micrographs of hepatocytes from LUL-flox and LUL-CKO administered Ad-ctrl or Ad-Lap1. Cells were stained with BODIPY (green) and DAPI (blue). A torsinA-deficient hepatocyte from an A-CKO mouse is shown for comparison. Scale bars: 10 μm. Proteins depleted from the cells are indicated at the bottom of each image.

Whole-liver lysates contain proteins from cells other than hepatocytes. We therefore isolated hepatocytes from livers of LUL-flox and LUL-CKO mice 5 days after injection of the adenoviral vectors to better assess the depletion of LAP1. Immunoblotting of isolated hepatocytes demonstrated diminished expression of all LAP1 isoforms in hepatocytes isolated from both LUL-flox and LUL-CKO mice administered shRNA Ad-Lap1; however, there was a greater decrease in LAP1A/B isoforms in LUL-CKO mice (Figure 4A). Quantification of total LAP1 signals on IBs of hepatocyte lysates showed that Ad-Lap1 treatment produced approximately 75% to 85% reductions in LAP1 expression in LUL-flox and LUL-CKO mice and a significant increase in torsinA expression (Figure 4B). The measured decreases in LAP1 expression and increases in torsinA expression in isolated hepatocytes were of greater magnitude than the changes measured in whole-liver lysates. While residual expression of LAP1 was detected by immunoblotting, we observed little to no nuclear rim labeling with anti-LAP1 Abs in confocal immunofluorescence micrographs of hepatocytes from both LUL-flox and LUL-CKO mice that received Ad-Lap1 (Figure 4C). We previously showed that torsinA depletion from hepatocytes leads to the accumulation of mostly microvesicular lipid droplets in the cytosol, whereas LAP1 depletion leads to nuclear lipid droplets with more modest cytosolic lipid accumulation (6, 20). We therefore stained hepatocytes with BODIPY and DAPI to analyze the subcellular localization of lipid droplets in those depleted of LAP1 and LULL1 (Figure 4D). While we observed relatively low numbers of normal-sized cytosolic lipid droplets in hepatocytes from LUL-flox and LUL-CKO mice treated with Ad-ctrl, there was increased cytosolic lipid and nuclear lipid droplets in hepatocytes from LUL-flox mice treated with Ad-Lap1 to deplete LAP1. In hepatocytes from LUL-CKO mice treated with Ad-Lap1, resulting in depletion of both LULL1 and LAP1, there was striking accumulation of small cytoplasmic lipid droplets, with minimal to no nuclear lipid droplets, similar to that in hepatocytes from A-CKO mice with depletion of torsinA (Figure 4D). When we stained these hepatocytes with anti-torsinA Ab and examined them by confocal immunofluorescence microscopy, we detected a more fragmented pattern of torsinA signal in the ER and stronger torsinA signal in the perinuclear region of hepatocytes that had been depleted of both LULL1 and LAP1 as compared with depletion of LULL1 alone (Supplemental Figure 8). This could be due to abnormal ER structure secondary to the numerous lipid droplets in the cytosol of cells with combined LULL1 and LAP1 depletion.

**Figure 5:**
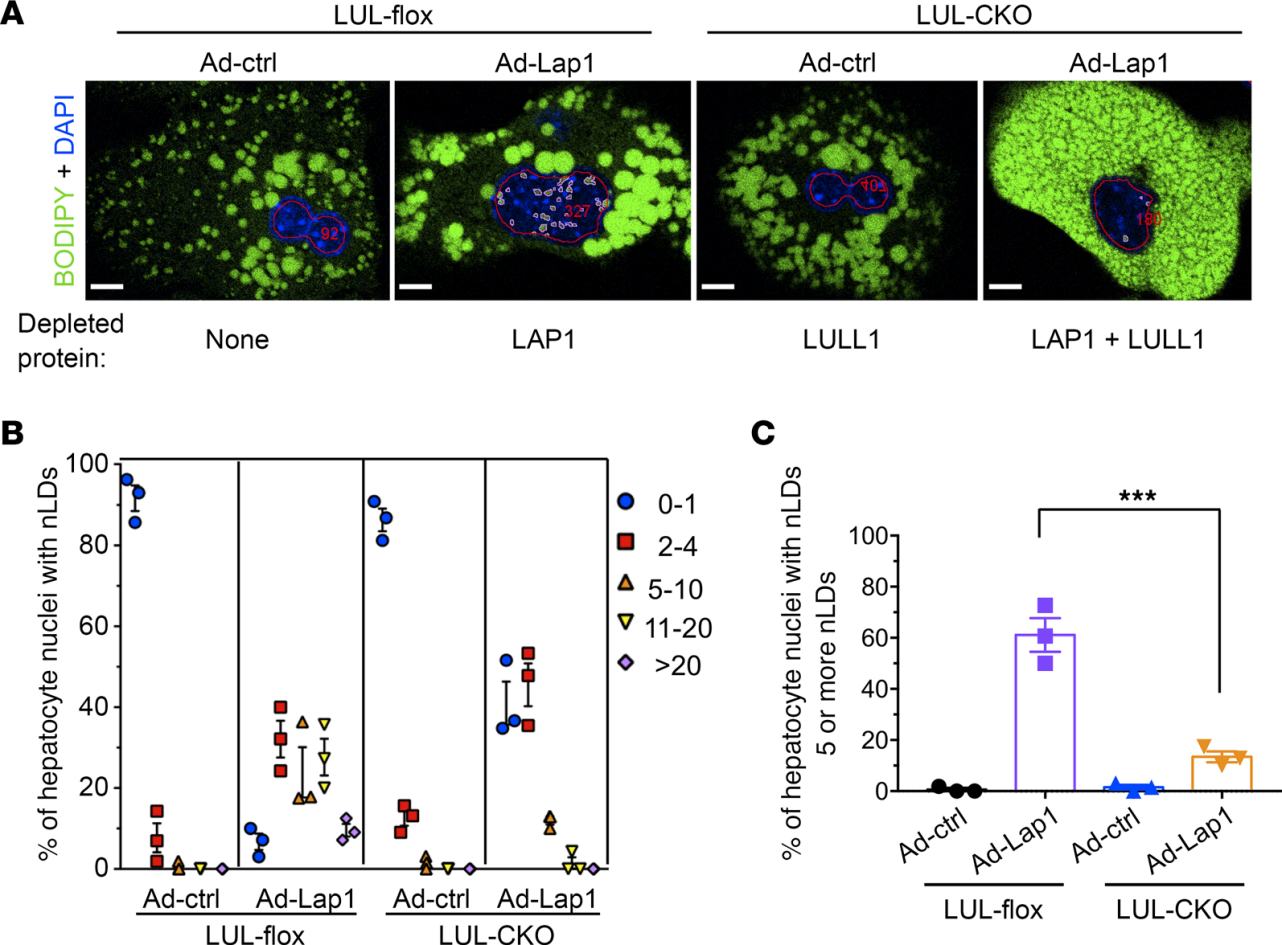
Nuclear lipid droplets in hepatocytes with depletion of LAP1, LULL1, or both. (**A**) Representative immunofluorescence confocal micrographs of BODIPY- (green) and DAPI-stained (blue) hepatocytes from LUL-flox and LUL-CKO mice injected with adenovirus expressing either Ad-ctrl or Ad-Lap1. Red lines mark nuclei and pink lines nuclear lipid droplets. Red numbers indicate the cell count according to the automated scoring method (see Methods). Scale bars: 10 μm. Proteins depleted from the cells are indicated at the bottom of each image. (**B**) Different-colored symbols represent the percentages of hepatocyte nuclei containing the indicated numbers of nuclear lipid droplets (nLDs). We analyzed a total of 131 (LUL-flox mouse administered Ad-ctrl), 101 (LUL-flox mouse administered Ad-Lap1), 136 (LUL-CKO administered Ad-ctrl), and 94 (LUL-CKO mouse administered Ad-Lap1) nuclei of hepatocytes cultured on 3 different coverslips (*n* = 3 per group). Values are mean ± SEM. (**C**) Percentage of hepatocyte nuclei containing more than 5 nLDs calculated from data shown in **B**. Values are mean ± SEM, with each circle or triangle representing the value from each coverslip (*n* = 3). ***P < 0.001 by 1-way ANOVA followed by Tukey’s post hoc test.

We performed additional confocal microscopic analyses to quantify nuclear lipid droplets in hepatocytes lacking LAP1, LULL1, or both (Figure 5A). While almost all hepatocytes with depletion of only LAP1 had more than 1 nuclear lipid droplet and with approximately 30% having 10 or more per nucleus, few hepatocytes with depletion of both LAP1 and LULL1 had more than 10 lipid droplets per nucleus (Figure 5B). In hepatocytes with depletion of only LAP1, we found that 61% ± 7% (mean ± SEM) of nuclei had 5 or more nuclear lipid droplets, whereas only 13.2% ± 2% hepatocytes with depletion of both LAP1 and LULL1 had 5 or more nuclear lipid droplets. (Figure 5C). These results indicate that depletion of LAP1 and LULL1 from hepatocytes produced the same subcellular distribution of lipid droplets that occurs in hepatocytes with depletion of torsinA (6).

### Depletion of both LAP1 and LULL1 from mouse hepatocytes leads to reduced liver VLDL secretion, similar to that occurring with torsinA depletion.

**Figure 6:**
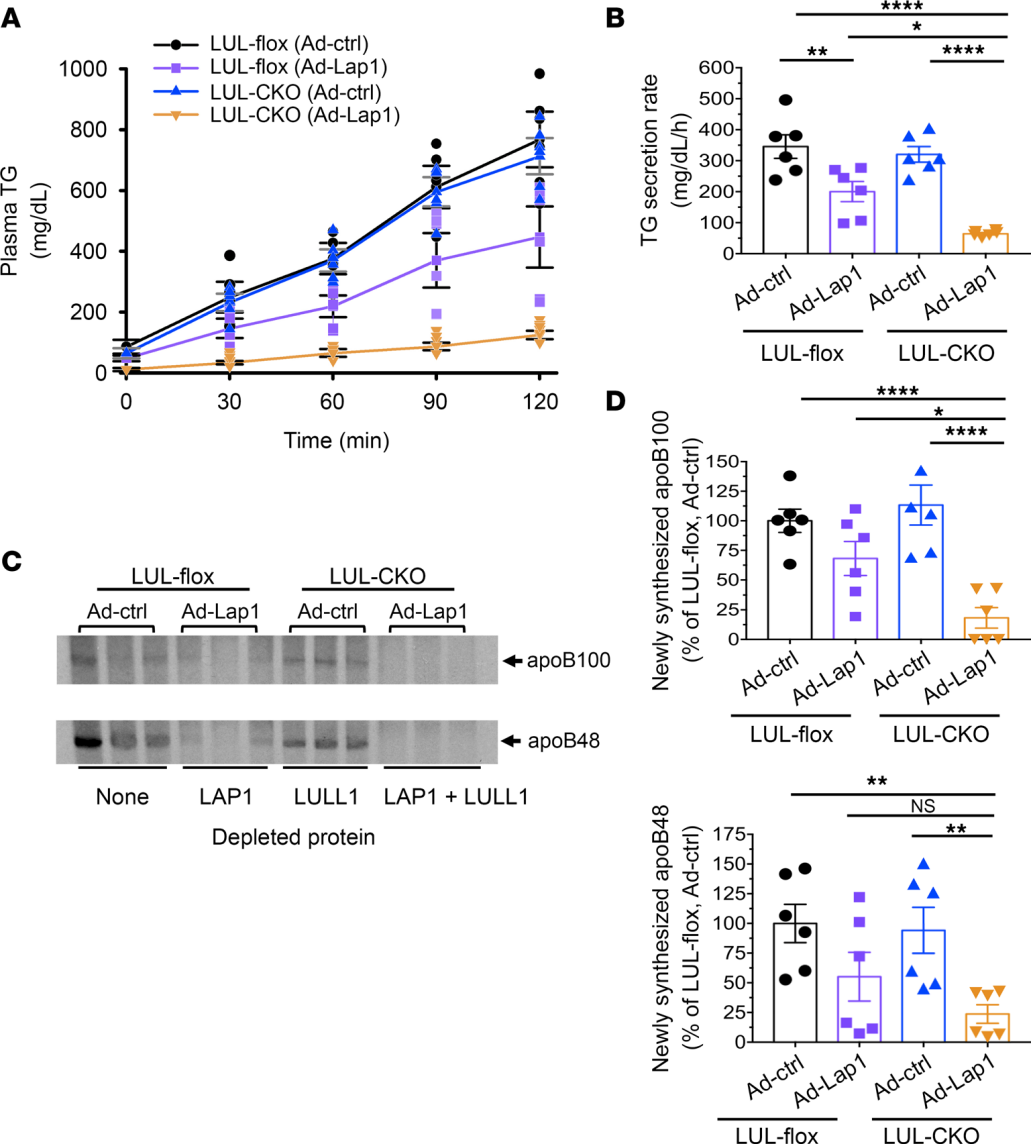
Depletion of LAP1 and LULL1 from hepatocytes leads to decreased liver TG secretion similar to that occurring with torsinA depletion. (**A**) Plasma TG concentration versus time after injection of tyloxapol to block peripheral uptake in LUL-flox and LUL-CKO mice 5 days after injection of adenoviral vectors expressing either control scrambled shRNA (Ad-ctrl) or shRNA that targets LAP1 (Ad-Lap1). Values are mean ± SEM, with each circle, square, or triangle representing the value for an individual mouse (*n* = 6). (**B**) TG secretion rates were calculated by changes in plasma concentration between the 30- and 120-minute time points in **A**. Values are mean ± SEM, with each symbol representing the value from an individual mouse (*n* = 6). *P < 0.05; **P < 0.01; ****P < 0.0001 by 1-way ANOVA followed by Tukey’s post hoc test. (**C**) Autoradiogram of SDS-polyacrylamide gel showing ^35^S-labeled plasma proteins collected 60 minutes after injection of mice with ^35^S-methionine. Each lane shows protein from an individual mouse (*n* = 3 per group). Migrations of ^35^S-labeled apoB100 and apoB48 are indicated. Proteins depleted from the cells are indicated at the bottom of the autoradiogram. (**D**) Bands corresponding to apoB100 (upper panel) and apoB48 (lower panel) from 2 different gel images obtained from 2 separate experiments were quantified by densitometry and shown as a percentage of the value from the Ad-ctrl–injected LUL-flox mice. Values are mean ± SEM, with each symbol representing the value from an individual mouse (*n* = 6). *P < 0.05; **P < 0.01; ****P < 0.0001 by 1-way ANCOVA with Tukey’s post hoc test to adjust for the experiment date as a covariate. NS, not significant.

We next tested whether hepatic steatosis in mice with hepatocytes depleted of both LAP1 and LULL1 is associated with defective VLDL secretion, as is the case in A-CKO mice with depletion of torsinA from hepatocytes (6). Due to the transient effect of adenovirus-mediated gene delivery systems, we performed in vivo TG secretion assays in LUL-flox or LUL-CKO mice 5 days after administration of either Ad-ctrl or Ad-Lap1. We measured plasma TG concentrations following administration of tyloxapol to block both lipolysis of VLDL-TG and uptake of circulating TG-rich VLDL remnant lipoproteins (Figure 6A). There were no significant changes in TG secretion rate in mice with no protein depleted (LUL-flox injected with Ad-ctrl) or only LULL1 depleted (LUL-CKO injected with Ad-ctrl) from hepatocytes; however, TG secretion was significantly reduced in mice with depletion of only LAP1 (LUL-flox mice injected with Ad-Lap1) and even further reduced in mice with combined LAP1 and LULL1 depletion (LUL-CKO mice injected with Ad-Lap1) (Figure 6B). Plasma samples collected 60 minutes after injection of mice with ^35^S-methionine and tyloxapol were analyzed by SDS-PAGE followed by autoradiography to detect radiolabeled apoB100 or apoB48 as a measure of their secretion (Figure 6C). Secretion of apoB100 in mice with depletion of both LULL1 and LAP1 from hepatocytes (LUL-CKO injected with Ad-Lap1) was significantly reduced compared with all other groups, including mice with hepatocyte depletion of only LAP1 (LUL-flox injected with Ad-Lap1) (Figure 6D). A similar pattern was observed for apoB48 secretion, except that the difference in secretion between LUL-CKO mice injected with Ad-Lap1 and LUL-flox mice injected with Ad-Lap1 did not reach statistical significance. Overall, our results indicate that mice with combined hepatic depletion of LAP1 and LULL1 accumulate large amounts of lipid in association with severe defects in VLDL secretion, which is similar to what we observed in A-CKO mice with depletion of torsinA from hepatocytes (6).

### Livers with combined depletion of both LAP1 and torsinA from hepatocytes show similar phenotypes to livers with depletion of only torsinA from hepatocytes.

If LAP1 is an activator of torsinA acting in the same pathway with regard to liver lipid metabolism, then combined depletion of both proteins from hepatocytes should generate similar liver phenotypes to those observed with depletion of only torsinA. We used the marked cytosolic lipid accumulation phenotype of torsinA-deficient hepatocytes and nuclear lipid droplet phenotype of LAP1-deficient hepatocytes as distinct readouts for the effects of combined depletion of both proteins. We did not include LULL1 in this analysis, as its deficiency from hepatocytes did not cause any readily apparent abnormal phenotype.

**Figure 7:**
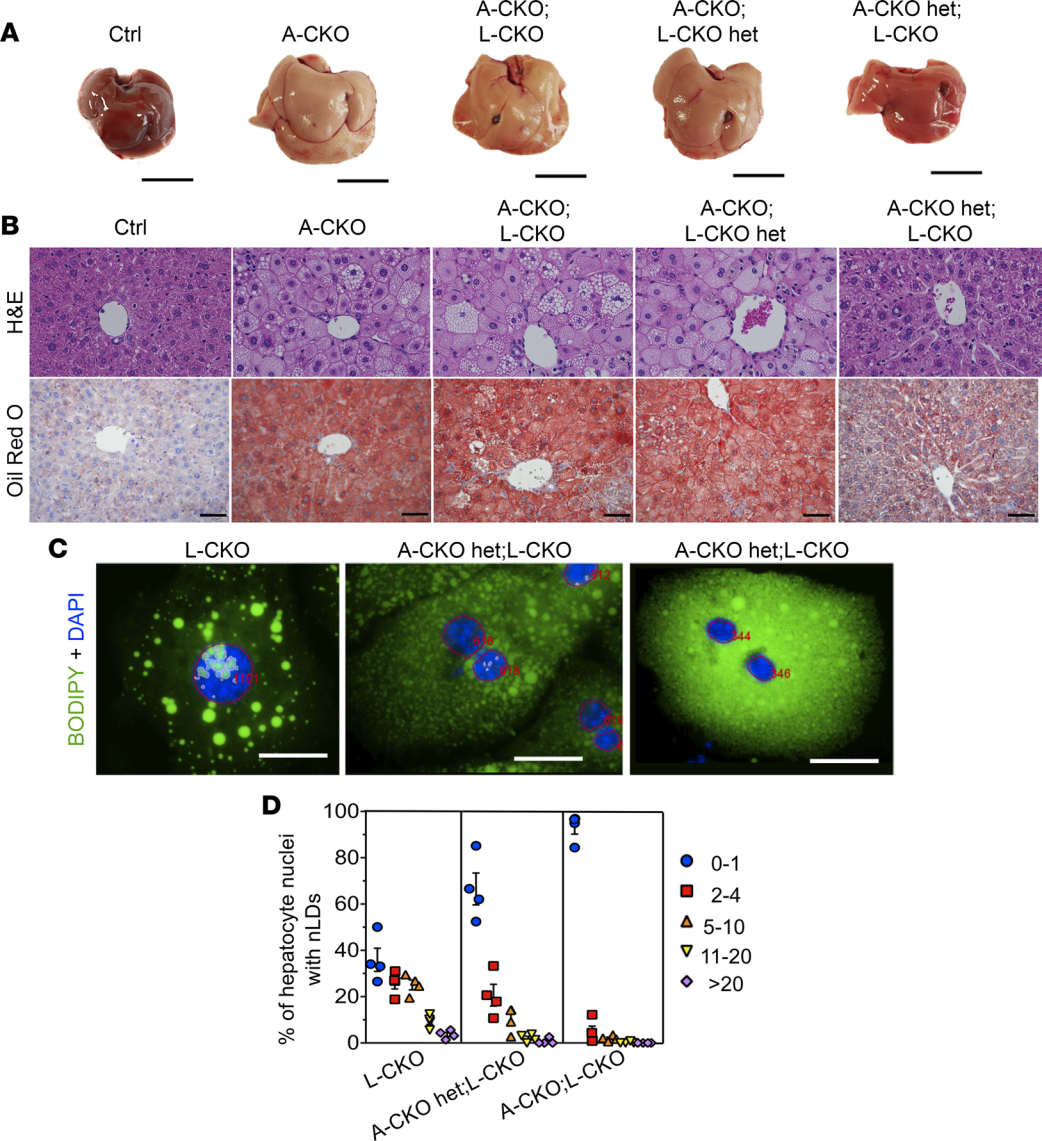
Combined depletion of LAP1 and torsinA from hepatocytes leads to phenotypes similar to that of depletion of torsinA alone. (**A**) Representative photographs of livers dissected from chow-fed mice with indicated genotypes. Scale bars: 1 cm. (**B**) Representative light photomicrographs of liver sections stained with H&E (upper panel) or Oil Red O (lower panel). Liver sections were prepared from control mice (Ctrl: *Tor1a^fl/fl^*;*Tor1aip1^fl/fl^* without Cre) and other genotypes shown in panel **A**. Scale bars: 50 μm. (**C**) Representative widefield fluorescence micrographs of BODIPY- (green) and DAPI-stained (blue) hepatocytes from mice with the indicated genotypes. Red lines mark nuclei and pink lines nuclear lipid droplets. Red numbers indicate the nuclear count according to the automated scoring method (see Methods). Scale bars: 25 μm. (**D**) Different-colored symbols represent the percentages of hepatocyte nuclei containing the indicated numbers of nuclear lipid droplets (nLDs). We analyzed a total of 494 (L-CKO), 317 (A-CKO het;L-CKO), and 576 (A-CKO;L-CKO) nuclei of hepatocytes cultured on 4 different coverslips (*n* = 4). The values are mean ± SEM.

We crossed A-CKO mice with mice with 2 floxed alleles of *Tor1aip1* encoding LAP1 to obtain mice with depletion of both torsinA and LAP1 from hepatocytes (A-CKO;L-CKO mice). We compared the phenotypes of these mice to A-CKO mice with depletion of only torsinA, A-CKO mice with deletion of only 1 *Tor1aip1* allele encoding LAP1 (A-CKO;L-CKO het), L-CKO mice with deletion of only 1 *Tor1a* allele encoding torsinA (A-CKO het;L-CKO), and control mice (Ctrl) with only floxed alleles of *Tor1a* and *Tor1aip1* but no Cre. We had shown previously that mice with depletion of only 1 *Tor1a* allele with approximately 50% reduction of torsinA do not develop steatosis (6). Mice of each of these genotypes were viable and their development was grossly normal. When we examined livers from these mice at 4–6 months of age, we found that A-CKO mice with either homozygous or heterozygous deletion of LAP1 had grossly white livers, similar to those of A-CKO mice with depletion of only torsinA (Figure 7A). Histological examination of H&E-stained or Oil Red O–stained liver sections of the indicated genotypes revealed that A-CKO mice combined with either homozygous or heterozygous deficiency of LAP1 showed similar degrees of liver steatosis compared to A-CKO mice (Figure 7B). A pathologist blinded to genotype gave the same steatosis score of 3 (on a scale of 0 to 3) to all sections examined from livers from A-CKO, A-CKO;L-CKO, and A-CKO;L-CKO het mice. In agreement with this histological assessment, the TG content of livers from A-CKO mice was similar to those in A-CKO;L-CKO and A-CKO;L-CKO het mice, all of which were significantly greater than control mice, and a trend toward increased TG in A-CKO het;L-CKO mice compared with control (Supplemental Figure 9). We next determined whether the frequency of nuclear lipid droplets in hepatocytes from L-CKO mice is altered by homozygous or heterozygous deficiency of torsinA. Isolated hepatocytes from L-CKO, A-CKO het;L-CKO, and A-CKO;L-CKO mice were stained with BODIPY and DAPI to identify nuclear lipid droplets. Fluorescence microscopy showed that the frequency of nuclear lipid droplets in hepatocytes from mice with combined LAP1 depletion and either homozygous or heterozygous depletion of torsinA were reduced compared with L-CKO mice with only LAP1 depletion (Figure 7C). In hepatocytes from L-CKO mice, we found that 64.5% (mean ± SEM) of nuclei contained 2 or more lipid droplets, whereas 33.7% of hepatocyte nuclei from A-CKO het;L-CKO mice and only 7.3 % from A-CKO;L-CKO mice contained 2 or more lipid droplets (Figure 7D). These results indicate that nuclear lipid droplet formation caused by LAP1 depletion from hepatocytes is suppressed by the loss of torsinA. We next examined liver TG secretion in A-CKO;L-CKO mice. A-CKO;L-CKO mice had reduced plasma TG concentrations following administration of tyloxapol (Supplemental Figure 10A). The TG secretion rate was significantly lower in A-CKO;L-CKO mice compared with controls (Supplemental Figure 10B). Accordingly, apoB100 secretion was significantly reduced compared with control mice (Supplemental Figure 10C), as was that for apoB48 (Supplemental Figure 10D). These results demonstrated that the depletion of both LAP1 and torsinA from hepatocytes produced similar liver phenotypes to those in A-CKO mice, suggesting an epistatic genetic interaction. Overall, our results demonstrate that LAP1 is a more critical activator of torsinA than LULL1 in hepatocytes, and that it functions in the same pathway with torsinA in hepatic TG secretion.

## Discussion

Several previous studies have demonstrated direct interactions between torsinA and LAP1 and between torsinA and LULL1 using various cell biological, biochemical, and structural approaches (10–12, 15). However, neither the physiological relevance of these interactions nor the relative contribution of the 2 activators to torsinA function in vivo has been demonstrated to our knowledge. We now show that combined depletion of LAP1 and LULL1 from hepatocytes leads to marked hepatic steatosis and VLDL secretion defects similar in magnitude to that occurring in A-CKO mice with depletion of torsinA from hepatocytes. These results provide compelling evidence that LAP1 and LULL1 both modulate torsinA function in controlling hepatocyte fat content in vivo.

LAP1 depletion from hepatocytes is associated with decreased VLDL section and steatosis; however, these are less severe than when torsinA is depleted (6). This led us to hypothesize that LULL1 can partially compensate for LAP1 in liver lipid metabolism. However, LULL1 depletion from hepatocytes does not cause hepatic steatosis or decreased VLDL secretion. The interaction of LAP1 and LULL1 with torsinA to enable its function in hepatocytes is therefore more complex than a binding to 2 different proteins, expressed at similar levels, with similar avidities. One possibility is that there is an unrecognized interaction with LAP1 that is essential for LULL1 to fully establish torsinA function. As LAP1 is a transmembrane protein of the inner membrane of the nuclear envelope, another possibility is that torsinA function in the perinuclear space is important for hepatocyte VLDL secretion. LULL1 is a monotopic transmembrane protein with a cytoplasmic domain of 215 aa (10). It is localized throughout the entire ER, which includes the outer nuclear membrane. Given the size of its cytoplasmic domain, some fraction of LULL1 could diffuse through the interconnected ER, outer nuclear membrane, pore membrane, and inner nuclear membranes (22). Therefore, in the absence of LAP1, some LULL1 could be localized to the nuclear envelope and may be responsible for providing the partial function of torsinA there. If so, it raises the question of why nuclear envelope–localized LAP1 appears to be the major activator of torsinA in hepatic lipid secretion despite LULL1 being localized to VLDL-forming components in the more peripheral ER.

The luminal regions of LAP1 and LULL1 contain a conserved domain where the arginine finger of canonical AAA+ ATPases is found (12, 23). The interaction of one of these activators with torsinA is predicted to generate an active site for ATPase activity. LAP1 or LULL1 binding to torsinA in vitro stimulates ATPase activity (11, 12). However, we cannot apply the assays used in vitro to liver or hepatocyte lysates because a substrate for torsinA enzymatic activity has not been identified. It therefore remains unknown whether ATPase activity of torsinA and its activators or some other process underlies their function in VLDL secretion and liver lipid metabolism.

A significant number of nuclei in hepatocytes with depletion of LAP1 contain nuclear lipid droplets, which are rarely observed with depletion of torsinA. Nuclear lipid droplets in hepatocytes with depletion of LAP1 likely arise from invaginations of the inner nuclear membrane, which are also known as type 1 nucleoplasmic reticula (20). Nuclear lipids associated with type 1 nucleoplasmic reticula also occur in hepatocellular carcinoma–derived cell lines and they are increased by depletion of SUNs, which like LAP1 are integral proteins of the inner nuclear membrane (24, 25). However, hepatocytes with combined depletion of LAP1 and LULL1 more closely resemble hepatocytes with depletion of torsinA, with few if any nuclear lipid droplets but numerous small and some larger cytoplasmic ones. Electron microscopy has shown that the numerous small lipid droplets in hepatocytes with depletion of torsinA appear to be arranged in tubular configurations, suggesting that they may be in the ER (6).

The combined depletion of LAP1 and torsinA from hepatocytes leads to a similar degree of lipid accumulation seen in hepatocytes with depletion of only torsinA. This suggests that torsinA is the major effector of lipid accumulation acting downstream of the activators. Moreover, our results showed that the combined depletion of torsinA and LAP1 from hepatocytes diminished the occurrence of nuclear lipid droplets significantly, which indicates an epistatic relationship between torsinA and LAP1 and implies that the 2 proteins work in the same pathway in hepatic lipid metabolism. These results are not well aligned with those suggesting that *Drosophila* torsinA and LAP1 act differentially in fat body lipid metabolism (26). The *Drosophila* fat body may differ, however, from the mammalian liver with regard to lipid biogenesis and regulation. Furthermore, the *Drosophila* genome does not have a gene encoding LULL1 and *dLap1* encoding LAP1 is not essential for fly development (26). In contrast, LAP1 is an essential protein in mouse and human development (27, 28). Future studies will be required to delineate the torsinA-dependent and torsinA-independent cellular functions of LAP1.

Our findings in mice support future studies on the precise roles of torsinA and its activators in human lipid metabolism and conditions such as SLD. A study in pigs has tentatively linked serum TG concentrations to polymorphisms in the gene encoding LAP1 (29). An intronic variant in *TOR1B* encoding torsinB, a paralogue of torsinA, has been linked to SLD in humans (30). Previous results suggest that torsinA and torsinB have redundant functions (11, 27). However, further research is necessary to determine whether there are different roles for these 2 proteins in human and mouse liver. Given the profound phenotypes we have observed in mice, polymorphisms or mutations in the genes encoding torsinA, LAP1, and possibly LULL1, likely influence hepatic lipid metabolism in humans. A more focused candidate gene approach may be necessary to prove a link to SLD in humans, as extremely rare variants with strong effects may be missed even in a large GWAS.

## Methods

### Mice.

We generated mice with hepatocyte-specific depletion of LULL1 by crossing female mice with homozygous floxed alleles of *Tor1aip2* encoding LULL1 with male Alb-Cre transgenic mice (Jackson Laboratory, stock 003574) (see Supplemental Figure 1). Genotyping was performed as shown in Supplemental Figure 1. Primer sequences are provided in Supplemental Table 1. L-CKO mice with depletion of LAP1 from hepatocytes, A-CKO mice with depletion of torsinA from hepatocytes, mice with floxed alleles of *Tor1a* encoding torsinA, and mice with floxed alleles of *Tor1aip1* encoding LAP1 have been previously described (6, 31). The genetic background of all mice was C57BL/6J. Mice were housed in a climate-controlled room with a 12-hour light/12-hour dark cycle and fed regular chow diet (Purina Mills, 5053). Both male and female mice were used for experiments, as our previous work showed no sex-specific differences in the phenotypes of A-CKO and L-CKO mice (6).

### Primary hepatocyte isolation.

Primary hepatocytes were isolated according to previously described methods (32). Briefly, mice were perfused with Hanks balanced salt solution without calcium (Thermo Fisher Scientific) with 8 mM HEPES (Thermo Fisher Scientific) via the abdominal inferior vena cava after cutting the portal vein to allow outflow of the perfusate. They were perfused for 8 minutes at a rate of 5 mL/min at 37°C. This was followed by perfusion at the same rate of DMEM (Thermo Fisher Scientific) with 80 mg/50 mL of collagenase type I (Worthington Biochemical) for 6 minutes. The liver was removed and minced in a Petri dish containing 4 mL of the same warm DMEM collagenase mixture for an additional 2 to 4 minutes. Ice-cold DMEM was added, and the digested tissue was filtered through a nylon mesh and collected in a 50 mL conical tube. The suspension was centrifuged for 5 minutes at 500 rpm. The supernatant was aspirated, and the cell pellet was washed 3 times with 30 mL of ice-cold DMEM. Viable cells were counted after staining with trypan blue and were greater than 90%. Isolated primary hepatocytes were used for experiments within 24 or 48 hours after isolation. In some cases, isolated hepatocytes from WT mice were frozen using Cryo-JIN solution (Revive Organtech), a specialized freezing media for primary hepatocytes, and thawed for immunofluorescent staining according to the manufacturer’s instructions.

### Cell culture.

C2C12 cells were purchased from ATCC (CRL-1772). Culture conditions were according to ATCC protocols. The isolated primary mouse hepatocytes were plated onto collagen-coated 6-well plates at a density of 500,000 cells/well in 4 mL of DMEM plus 10% FBS, 1% penicillin-streptomycin (Thermo Fisher Scientific), and 1% HEPES, and cultured for 18 to 48 hours for subsequent experiments, including immunoblotting and apoB secretion assays.

### Generation of adenoviral vectors and administration to mice.

To generate an adenoviral vector expressing shRNA targeting LAP1, 4 different shRNA sequences targeting mouse LAP1 were synthesized and cloned into a pSuper-retro-puro plasmid (Oligoengine) according to the manufacturer’s instructions. Knockdown efficiency was tested by transducing retroviral constructs onto C2C12 cells and immunoblotting lysates. After choosing the sequence showing most efficient knockdown (CAAGTGTCTGAGTGAACAAAT corresponding to a portion of *Tor1aip1* exon 10), a synthesized oligonucleotide was inserted downstream of the *U6* promoter in an adenoviral vector, and virus was packaged, amplified, and purified (Welgene). A control adenoviral vector expressing a scrambled shRNA under control of the same promoter was purchased from Welgene. Adenovirus was delivered to 3- to 6-month-old mice at a dose of 1 × 10^9^ to 2.5 × 10^9^ PFU/kg by retro-orbital injection.

### Immunoblotting.

Livers and cultured cells were homogenized in cell lysis buffer (Cell Signaling Technology) supplemented with 0.1% SDS, 0.2% sodium deoxycholic acid, Protease Inhibitor Cocktail (Sigma-Aldrich), and 1 mM phenylmethylsulfonyl fluoride (Sigma-Aldrich). Proteins in the samples were denatured by boiling in Laemmli sample buffer (33) containing β-mercaptoethanol for 5 minutes, resolved by 10% or 4% SDS-PAGE, and transferred to nitrocellulose membranes. For immunoblotting, membranes were washed with blocking buffer (Tris-buffered saline [TBS] containing 5% nonfat dry milk and 0.1% polysorbate-20) for 30 minutes and then probed with primary Abs in blocking buffer overnight at 4°C. Primary Abs were against LAP1 (10), LULL1 (10), torsinA (Abcam, ab34540), β-actin (Santa Cruz Biotechnology, sc-47778), myosin heavy chain (Santa Cruz Biotechnology, C-20641), γ-tubulin (Sigma-Aldrich, T5326), and GAPDH (Ambion, AM4300). Membranes were washed with TBS containing 0.1% polysorbate-20 and then incubated in blocking buffer containing horseradish peroxidase–conjugated secondary Abs (GE Healthcare) for 1 hour at room temperature. After washing with TBS containing 0.1% polysorbate-20, membranes were soaked with Enhanced Chemiluminescence substrate (Thermo Fisher Scientific) and exposed to x-ray films. To quantify signals, the exposed films were scanned, and the densities of the bands were quantified using ImageJ software (34).

### Histopathology.

Livers were immediately excised and blotted dry after euthanizing mice. Portions of excised livers were fixed in 10% formalin for 48 hours. Paraffin block preparation, sectioning into 5 μm slices, and staining with H&E were performed by the Histology Service of the Columbia University Molecular Pathology Core. Other portions of livers were transferred to a 30% sucrose solution and frozen sections 5 μm think were prepared by the Histology Service of the Columbia University Molecular Pathology Core for staining with Oil Red O (Polyscience, 25962). Stained sections were photographed using a BX53 upright microscope attached to a DP72 digital camera (Olympus). A liver pathologist blinded to genotype or treatment analyzed the H&E-stained sections and provided a steatosis score from 0 (<5% of parenchymal involvement) to 3 (>66%), considering total macrovesicular and microvesicular fat.

### Quantification of liver TG.

Liver lipids were extracted with a modified Folch method as previously described (35). Briefly, a snap-frozen piece of liver (~100 mg) was homogenized in PBS and lipids were first extracted with chloroform/methanol (2:1 v/v) and a second time with chloroform/methanol/water (86:14:1 v/v/v). The organic layer was dried under nitrogen gas and resolubilized in chloroform with 15% Triton X-100 (Sigma-Aldrich). It was dried under nitrogen gas and finally dissolved in double-distilled water.

### In vivo TG and apoB secretion.

In vivo TG and apoB secretion rates were determined as previously described (32). Briefly, mice were fasted for 4 to 5 hours prior to an intravenous injection of a mixture of 200 μCi ^35^S-methionine and 500 mg/kg tyloxapol (Sigma-Aldrich, T8761-50G) in 0.9% NaCl. Tyloxapol inhibits both the lipolysis and tissue uptake of lipoproteins in mice, and the subsequent concentrations of TGs and ^35^S-apoB in plasma can be used to calculate their rates of secretion. Blood samples were collected before injection and 30, 60, 90, and 120 minutes after injection of tyloxapol and ^35^S-methionine. Plasma TG concentrations were measured using a colorimetric assay (Wako Diagnostics, 461-08992). For apoB secretion, whole plasma samples from the 60-minute time point were resolved by 4% SDS-PAGE. The volumes of the samples were adjusted as determined by trichloroacetic acid–precipitable ^35^S-labeled plasma proteins. The gel was dried and exposed to x-ray film to quantitate labeled apoB proteins by densitometry.

### Fluorescence microscopy.

For fluorescence microscopy of isolated hepatocytes, cells were seeded onto collagen-coated coverslips in 6-well plates (150,000 cells/well) and grown overnight in a 37°C and 5% CO_2_ incubator before fixation or lipid staining. Cultured primary hepatocytes on collagen-coated cover slips were stained with 2 μM BODIPY (Thermo Fisher Scientific, D-3922) in Hank’s balanced salt solution for 30 minutes. Subsequently, cells were fixed with 4% paraformaldehyde for 15 minutes at room temperature, and then washed and stained with DAPI. For Ab staining, the fixed cells were incubated with primary Abs overnight. Primary Abs used were anti-LAP1 (10), anti–lamin A/C (Abcam, ab133256), and anti-torsinA (Abcam, ab34540). After incubation with primary Abs, cells were washed and then labeled with Alexa Fluor 488–conjugated goat anti-mouse and rhodamine-conjugated goat anti-rabbit secondary Abs (Jackson ImmunoResearch Laboratories). Confocal micrographs were obtained using a TCS SP8 DLS microscopy (Leica) in the Medicine Microscopy Core at Columbia University Irving Medical Center. For images shown in Figure 2D, we used an A1 RMP microscope using NIS-elements software (Nikon) at the Confocal and Specialized Microscopy Shared Resource of the Herbert Irving Comprehensive Cancer Center at Columbia University Irving Medical Center. Acquired fluorescence images were processed using NIS-elements software, ImageJ (NIH), or Fiji (34).

For automated analysis of fluorescence images, each batch of hepatocytes was plated on 3–4 coverslips and lipid droplets stained with BOPIDY 493/502 (Thermo Fisher Scientific) according to the manufacturer’s instructions. DAPI was included in the media for nuclear staining. Fluorescence images from 5–10 different regions were captured. Cytosolic and nuclear lipid droplets were quantified using macro programs created in Fiji/ImageJ (34). For confocal images, DAPI-stained nuclei were subject to Gaussian blur and triangle thresholding, and resulting nuclear objects were reduced in size by 1.8 μm to exclude cytosolic florescence. BODIPY-stained lipid droplets within nuclear objects were subject to a spot enhancing filter (36), Gaussian blur filter, and triangle threshold to create droplet objects. Nuclei and lipid droplet object outlines were overlaid on fluorescence images, cells were counted, parameters were measured, and a data table was created containing nuclear area, nuclear region, nuclear lipid droplet area, and nuclear lipid droplet BODIPY fluorescence intensity. For cytosolic fluorescence measurements, the analysis macro was modified to include object creation of individual cells using the deep learning software Cellpose (37), which was run within ImageJ. Nuclear objects found to reside within the cytosol were subtracted from the cytosol objects using the XOR function within ImageJ ROI manager and cytosol area and BOPIDY intensity were output to a table.

### Statistics.

Unpaired, 2-tailed Student’s *t* test was used to compare differences between any 2 groups (GraphPad Prism, version 9.4.0). Comparisons of more than 2 groups were performed by using 1-way ANOVA with a subsequent Tukey post-hoc test. Before proceeding with ANOVA, the fundamental assumptions of normality and homoscedasticity were assessed to ensure the validity of the statistical tests. The normality assumption was assessed using the Shapiro-Wilk test, with a *P* value of less than 0.05 indicating a violation of normality. The assumption of homoscedasticity was evaluated using Levene’s test, with a *P* value of less than 0.05 indicating the presence of heteroscedasticity. If all data met the assumptions of normality and homoscedasticity, then 1-way ANOVA with Tukey’s post hoc test was applied to identify specific pairwise differences between the group means. For Figure 6D, we conducted the statistical analysis on the experiment data pooled from 2 separate experiments on 2 different dates. ANCOVA with Tukey’s post hoc test was therefore conducted to adjust for the experiment date as a covariate. Statistical analyses for multiple group comparisons were performed using Statistical Analysis Software, version 9.4M6 (SAS Institute Inc). A significance level of α equal to 0.05 was adopted to determine the statistical significance of the results.

### Study approval.

The IACUC at Columbia University approved all procedures conducted in accordance with the NIH *Guide for the Care and Use of Laboratory Animals* (National Academies Press, 2011).

### Data availability.

All data generated and analyzed in this study are included in the article, in the supplemental Supporting Data Values file, and are available from the authors upon request.

## Author contributions

WTD, HNG, HJW, and JYS conceived of the project. AHO, WTD, HNG, HJW, and JYS designed experiments. WTD generated mouse lines. JYS designed and generated the viral construct. AHO, YPZ, JWM, CÖ, MJL, and JYS performed experiments. MJL analyzed the H&E-stained sections and provided steatosis scores. AHO, YPZ, JWM, AS, HNG, HJW, and JYS analyzed data. WTD, HNG, HJW, and JYS obtained funding. AHO, YPZ, WTD, HNG, HJW, and JYS wrote the manuscript. All authors reviewed and contributed to the final manuscript. The co-first authors AHO and YPZ are listed in alphabetical order by last name.

## Supplementary Material

Supplemental data

Unedited blot and gel images

Supporting data values

## Version 1 01/09/2024

In-Press Preview

## Version 2 02/08/2024

Electronic publication

## Version 3 05/13/2025

Ji-Yeon Shin's present address was added
