# Supplementary material for: Dynamic regulation of hepatic lipid metabolism by torsinA and its activators
Source: JCI Insight. 2024 Jan 9;9(3):e175328. doi: 10.1172/jci.insight.175328 (PMC10967386; doi:10.1172/jci.insight.175328)
Supplement: Supplemental data [file jciinsight-9-175328-s008.pdf]

Supplemental Material

Methods for Supplemental Material

Real-time quantitative PCR. Total RNA was extracted from dissected livers from mice 4-6 mo of age using the RNeasy Isolation Kit (Qiagen). Quality and concentrations of RNA were measured using a Nanodrop spectrophotometer (Thermo Fisher Scientific). The cDNA was synthesized using Superscript First Strand Synthesis System instructions (Thermo Fisher Scientific). For each replicate in each experiment, RNAs from livers of different animals were used (*n* = 4 per group). The sequences of quantitative PCR primers were either designed using Primer3 software or used with validated primers from the PrimerBank (1). PCR was performed on a MX3005p qPCR System (Stratagene) using Brilliant III Ultra Fast SYBR Green qPCR Master Mix (Agilent Technologies, 600883). Relative levels of mRNA expression were calculated using the $\Delta\Delta$CT method (2). Individual expression values were normalized by comparison to mRNA for HRPT.

Fluorescence microscopy of liver sections. For immunofluorescence microscopy of liver sections, liver tissues were fixed in 10% formalin for 24h, then fixed tissues were embedded in OCT compound as a frozen block before sectioning. Liver sections were post-fixed in 4% paraformaldehyde for 5 min at room temperature, then washed with PBS 3 times. Sections were then subjected to treatment with Antigen Unmasking Solution (Vector Laboratory, #H-3300) according to the manufacturer's instructions. The treated sections were incubated with blocking solution (5% BSA, 2% normal goat serum, and 0.5% Triton X-100 in PBS) for 30 min at room temperature and subsequently incubated with anti-LAP1 Ab (10) in the blocking solution overnight at 4°C. After 3 wash with PBS containing 0.1% polysorbate-20, the sections were incubated with Alexa fluor 488 conjugated secondary Abs (Thermo Fisher Scientific) with DAPI for 1 h at room temperature. After 3 additional washes, sections were mounted with ProLong mounting media (Thermo Fisher Scientific). Widefield fluorescent micrographs were acquired using a BX53 upright fluorescent microscope with an attached DP72 digital camera (Olympus).

Co-IP of LAP1 and LULL1 with torsinA. Recombinant adenovirus expressing flag only or flag-torsinA were generated using AdEasy Adenoviral Vector system according to the manufacturer's instruction (Agilent). Briefly, a flag-torsinA expression cassette in pCDNA3 plasmids was subcloned into a pAd-shuttle vector. Subsequently, the shuttle vector expressing the flag-torsinA cassette was co-transformed with pAdEasy-1 plasmid into BJ5183 to generate a recombinant adenovirus. The recombinant adenoviral vectors were packaged in AD-293 cells. Adenoviral amplification and purification steps were performed in Welgene. Primary hepatocyte cultures in 10-cm plates transduced with 10 MOI of the adenoviral vectors were harvested and resuspended in 2 ml IP buffer (400 mM NaCl, 0.5% Nonidet P-40, 0.1 % Triton-X-100, 5% glycerol, 50 mM Tris-HCl, pH 7.4) and homogenized in a Dounce homogenizer. Insoluble materials were removed by centrifugation at 16,000g for 20 min at 4°C and pre-cleared lysates were incubated with anti-flag M2 Ab conjugated to magnetic beads (Sigma-Aldrich, #M8823) for 4 h at 4°C. After incubation, the magnetic beads with Abs were washed with IP buffer 3 times, and bound materials were eluted by boiling in Laemmli sample buffer (3) containing β-mercaptoethanol for 5 min. Protein inputs (1% of total lysate) and eluted products were separated by SDS-PAGE, transferred to nitrocellulose, and probed with Abs against flag, torsinA, LAP1 and LULL1 as indicated.

Automated quantification of torsinA labeling in cells. For quantification of torsinA distribution, images of DAPI and torsinA stained primary hepatocytes were subject to Fiji/ImageJ background subtraction (rolling ball, radius 10) followed by quantification using CellProfiler (4). In brief, a CellProfiler pipeline (Hernandez-Ono_TorsinA_Quantification.cpproj) was written that identified nuclei, allowing multi-nuclei doublets to remain as single objects, identified cytoplasm as the detectable cell region above the background seeded by the nuclei, then excluded the nuclei from this mask, first subtracting 3 pixels (0.67 μm) from the edge of the nucleus to include the nuclear envelope. Intensity and other parameters of this mask were measured in the torsinA channel.

Plasma glucose and Insulin measurements. Plasma samples from mice were collected after 4 h of fasting. Glucose concentration was measured by an enzymatic method using a commercial kit

(Fujifilm Healthcare Americas Corp Autokit Glucose 99703001). Plasma insulin concentrations were measured using the Mouse Insulin ELISA kit (Crystal Chem 90080).

Supplemental References

1. Wang X, et al. Primerbank: A pcr primer database for quantitative gene expression analysis, 2012 update. *Nucleic Acids Res*. 2012;40(Database issue):D1144-D1149.
2. Ponchel F, et al. Real-time pcr based on sybr-green i fluorescence: An alternative to the taqman assay for a relative quantification of gene rearrangements, gene amplifications and micro gene deletions. *BMC Biotechnol*. 2003;3:18.
3. Laemmli UK. Cleavage of structural proteins during the assembly of the head of bacteriophage T4. *Nature*. 1970;227, 680-685.
4. Stirling DR, et al. CellProfiler 4: improvements in speed, utility and usability. *BMC Bioinformatics*. 2021;22:433.

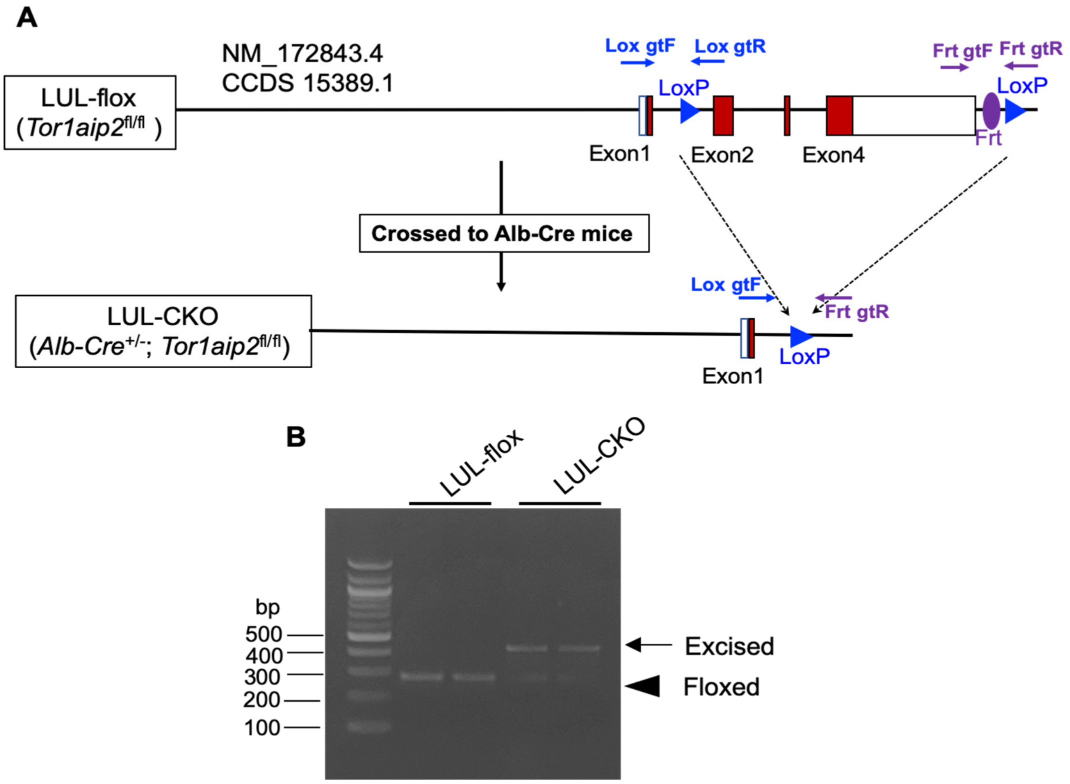

Supplemental Figure 1. Generation of LUL-CKO mice. (**A**) To generate LUL-flox mice, the *Tor1aip2* locus (NM_172843.4 or CCDS 15389.1) encoding LULL1 was targeted and engineered to have two loxP sequences (blue triangle) flanking exon 2 to exon 4 (exons shown in red) and a Frt sequence (purple circle) after the end of 3' UTR region of the gene. Blue arrows indicate primers Lox gtF and Lox gtR to detect the first LoxP site located in an intron between exon 1 and exon 2. Purple arrows indicate primers Frt gtF and Frt gtR to detect the Frt sequence. The sequences of corresponding primers are listed in the Supplemental Table 1. To generate LUL-CKO mice with depletion of LULL1 from hepatocytes, we crossed the LUL-flox mice to Alb-Cre transgenic mice to excise the LoxP flanking region. (**B**) SYBR Green-stained agarose gel showing PCR products demonstrating Cre-mediated DNA excision in mouse livers. Genomic DNA was extracted from livers of LUL-flox and LUL-CKO mice. PCR was performed using primers (Lox gtF, Frt gtF and Frt gtR) that detect the floxed (253 bp, arrow head) and excised (420 bp, arrow) alleles. Both floxed and excised alleles were detected from LUL-CKO samples due to the presences of other non-hepatocyte cells. Molecular mass standards (DNA ladder) are shown in the leftmost lane of the gel.

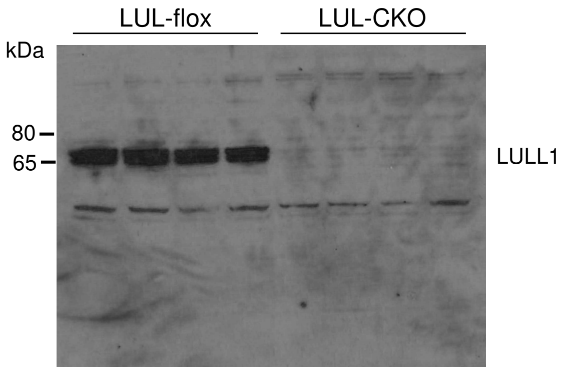

Supplemental Figure 2. Overexposed IB of liver lysates from LUL-flox and LUL-CKO mice probed with Abs against LULL1. Each lane is a sample from a different mouse. The IB is the same as that shown in Figure 1C of the main text exposed for a longer period of time.

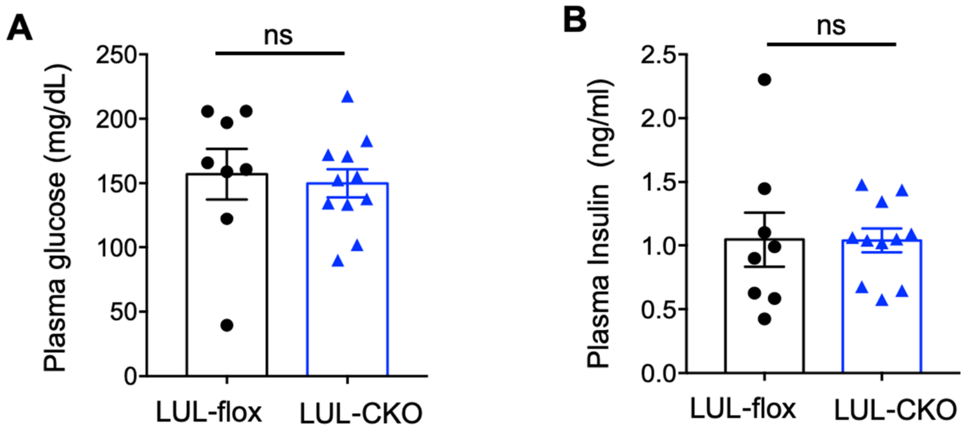

Supplemental Figure 3. Plasma glucose and insulin concentrations in LUL-CKO mice. (**A**) Plasma glucose concentrations. Mice were fasted for 4 h before collecting plasma. (**B**) Plasma insulin concentrations. Mice were fasted for 4 h before collecting plasma. Values are means ± SEM with each circle or triangle representing the value from an individual mouse. ns = not significant by Student's t-test.

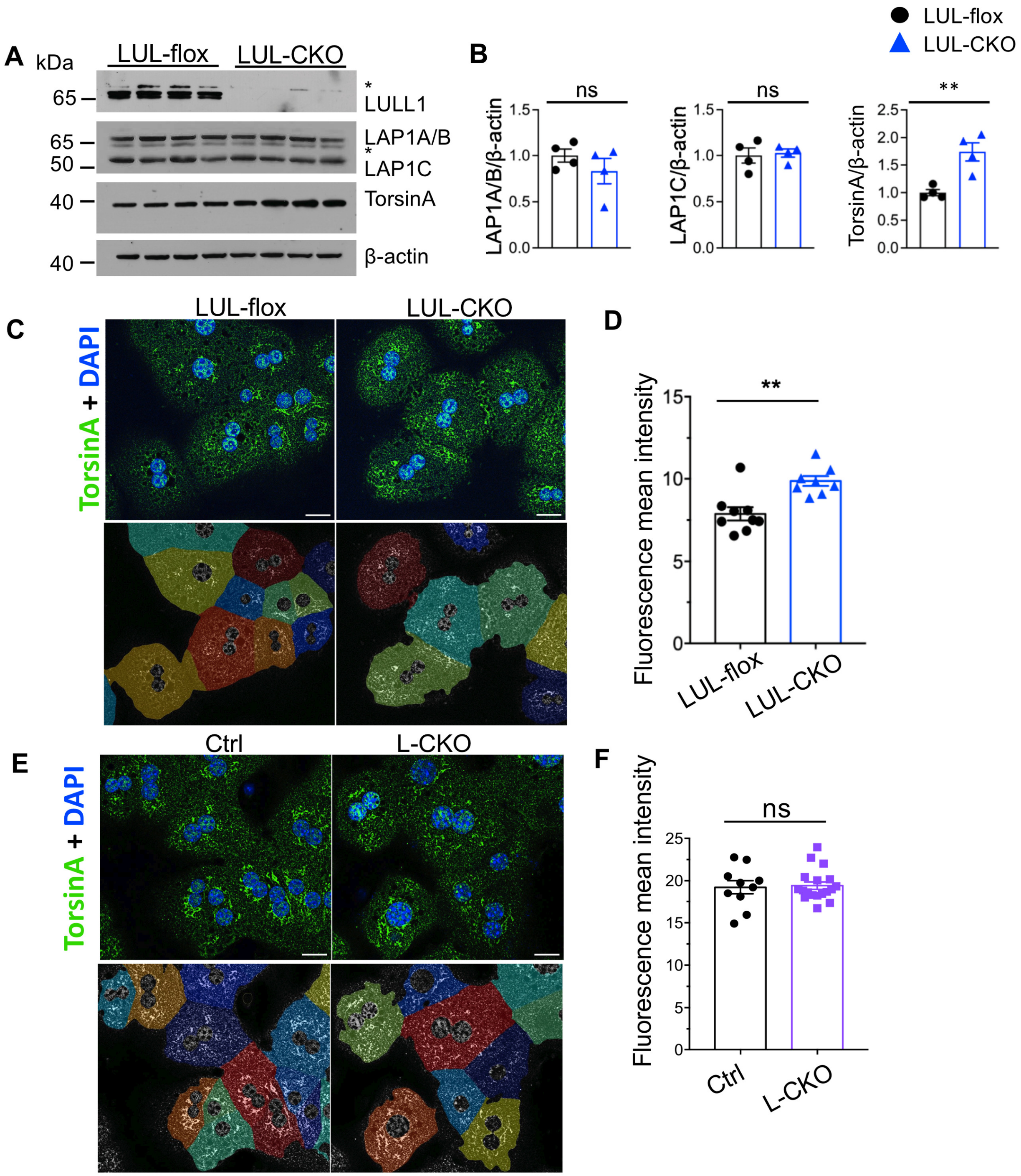

Supplemental Figure 4. Modest increase of torsinA expression in livers from LUL-CKO mice and normal torsinA distribution in hepatocytes isolated from either LUL-CKO or L-CKO mice. (**A**) IBs of liver lysates from LUL-flox and LUL-CKO mice at 4-6 mo of age. Blots were probed with Abs against LULL1, LAP1, torsinA, and β-actin. The anti-LULL1 ab detects two closely migrating bands (see reference 14 in main article). Mice express three LAP1 isoforms — LAP1A, LAP1B, and LAP1C — with

LAP1A and LAP1B varying by only 19 amino acids. *Indicates non-specific bands often detected in whole liver lysates using polyclonal Abs against LAP1 or LULL1. Each lane is a lysate from an individual mouse. (**B**) Relative ratios of band densities of LAP1A/B, LAP1C, or torsinA to β-actin blots are shown in panel A (n = 4 mice per group). Band densities were measured from IBs using protein extracts from four mice per group. Values are means ± SEM with each circle or triangle representing the value from each lane of the IB shown in panel A. ns = not significant, $^{**}P < 0.01$ by Student's t-test. (**C**) Immunofluorescence confocal microscopic images of primary hepatocytes isolated from LUL-flox and LUL-CKO mice. Cells were stained with anti-torsinA Ab (green) and DAPI (blue, upper panel). TorsinA mask images for individual cells (indicated with different colors) from the confocal micrographs shown in the upper panel were run through a CellProfiler automated image analysis pipeline (bottom panel). Cytosolic torsinA fluorescence mean intensity was quantified using this mask. Scale bars: 20 μm. (**D**) Cytosolic torsinA fluorescent mean intensity values were plotted from 8-9 different images of a total of 153 hepatocytes from LUL-flox and 100 hepatocytes from LUL-CKO mice. (**E**) Immunofluorescence confocal micrographs of primary hepatocytes isolated from (Lap1-flox: Ctrl) and L-CKO mice. Cells were stained with anti-torsinA Ab (green) and DAPI (blue, upper panel). TorsinA mask images for individual cells (indicated with different colors) from the confocal images shown in the upper panel were run through a CellProfiler automated image analysis pipeline (bottom panel). Cytosolic torsinA fluorescence mean intensity was quantified using this mask. Scale bars: 20 μm. (**F**) Mean fluorescence intensity signals were calculated from 10 different images of a total 142 hepatocytes from Lap1-flox and 18 different images of a total of 353 hepatocytes from L-CKO mice. In panels in D and F, data are means ± SEM. Circles, triangles and squares indicate the mean intensity values of each image. $^{**}P < 0.01$, ns = not significant by Student's t-test.

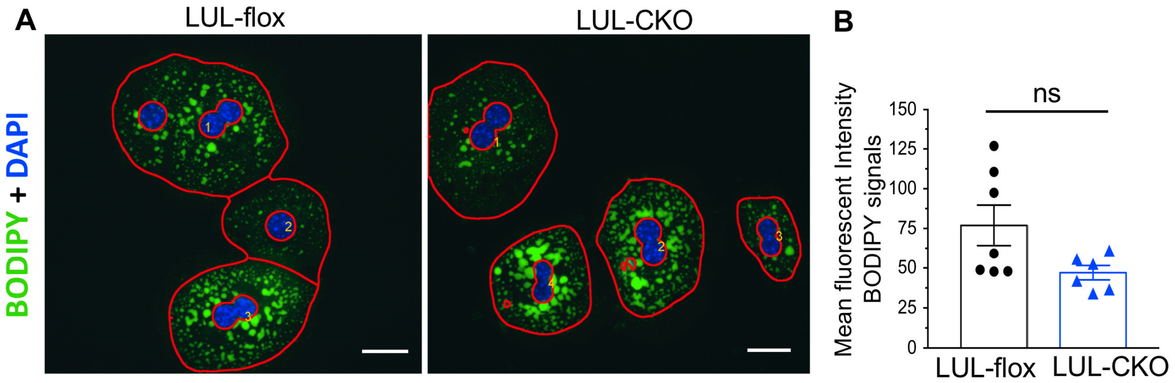

Supplemental Figure 5. Normal lipid droplet distribution in hepatocytes isolated from LUL-CKO mice. (**A**) Confocal micrographs of primary hepatocytes from LUL-flox and LUL-CKO mice stained with BODIPY and DAPI. The red lines marked cell boundary and excluded the nuclear region for measurement of cytosolic fluorescent intensity. Numbers with white color indicate counting of the cells during automated scoring (see Methods). Scale bars: 20 µm. (**B**) Mean fluorescence intensity plots of BODIPY signals were calculated from 7 different images of a total 48 hepatocytes from LUL-flox and 6 different images of a total of 35 hepatocytes from LUL-CKO mice. Data are means ± SEM. Circles and triangles indicate the mean intensity values of each image. ns = not significant by Student's t-test.

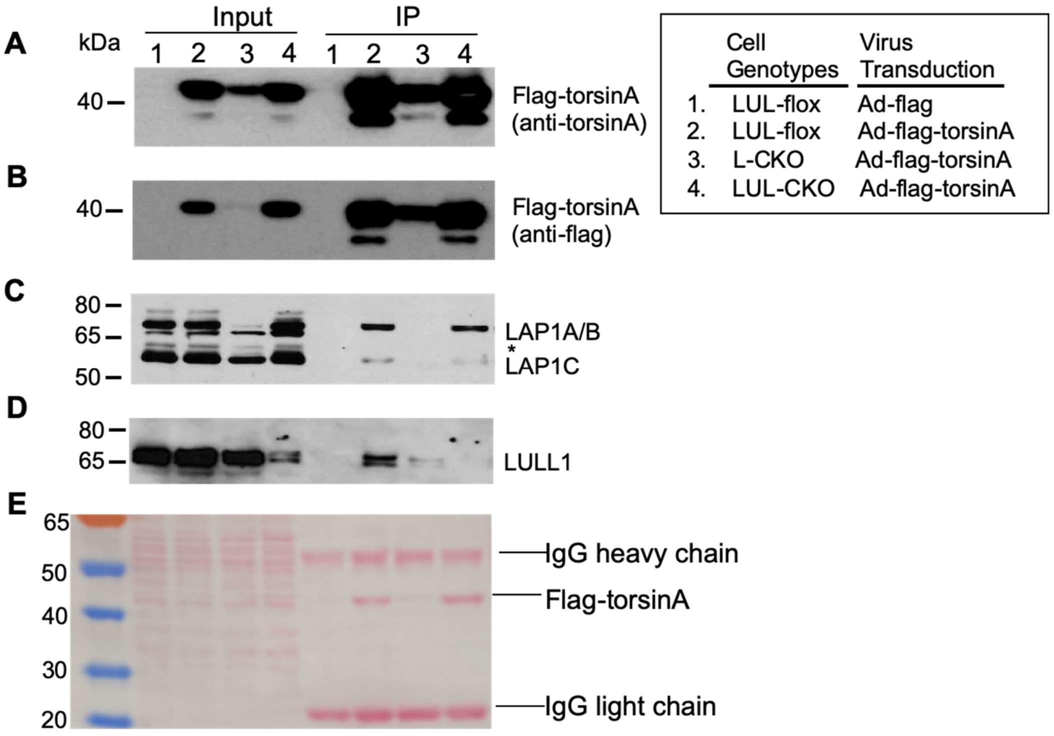

Supplemental Figure 6. TorsinA interacts with its activators in mouse hepatocytes. Box at upper right is key for cell genotype and virus transduction for result shown in lanes 1-4 in subsequent panels that created the following hepatocytes: 1) control cells expressing flag, 2) control cells expressing flag-torsinA, 3) cells with depletion of LAP1 expressing flag-torsinA, 4) cells with depletion of LULL1 expressing flag-torsinA. Co-IP was performed with anti-flag Abs conjugated to magnetic beads. (**A**) IB shows flag-torsinA in input (1% of total cell lysates used for IP) and co-precipitated products in four different samples with genotypes and treatments as indicated in the box. The blot was probed with anti-torsinA Ab. (**B**) IB of the same samples used in (A) probed with anti-flag Ab to verify flag-torsinA expression. (**C**) IB similar to those in (A and B) probed with anti-LAP1 Ab that detects isoforms of LAP1 (*indicates non-specific band). The blot shows that IP of flag-torsinA co-precipitated endogenous LAP1 in lysates of control hepatocytes (lane 2) and hepatocytes with depletion of LULL1 (lane 4). Co-IP of LAP1 was not observed in control hepatocytes expressing only flag (lane 1) or hepatocytes with depletion of LAP1 expressing flag-torsinA (lane 3). Some LAP1 was detected in the input of cell lysates from L-CKO mice, possibly originating from liver cells other than hepatocytes. (**D**) IB similar to those in

(A, B, C) probed with anti-LULL1 Ab. The blot shows that IP of flag-torsinA co-precipitated endogenous LULL1 in lysates of control hepatocytes (lane 2) and hepatocytes with depletion of LAP1 expressing flag-torsinA (lane 3). Co-IP of LAP1 was not observed in control hepatocytes expressing only flag (lane 1) or hepatocytes with depletion of LULL1 expressing flag-torsinA (lane 4) Some LULL1 was detected in the input of cell lysates from LUL-CKO mice, possibly originating from liver cells other than hepatocytes. (**E**) Representative Ponceau-stained nitrocellulose membrane showing equal amount of protein loading among input samples. IgG heavy and light chains were eluted together in samples subjected to co-IP after boiling in Laemmli sample buffer.

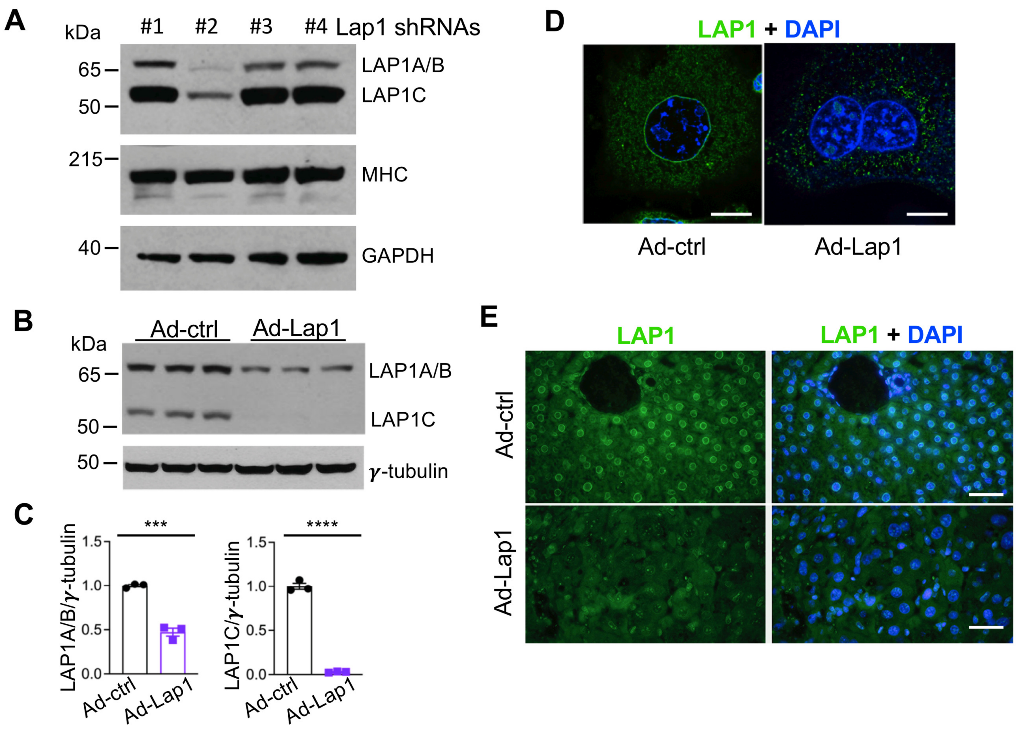

Supplemental Figure 7. Validation of adenoviral vectors expressing shRNA to deplete LAP1 from hepatocytes. (**A**) IBs of C2C12 cell lysates collected 48 hours after transduction of retroviral constructs expressing four different shRNAs that target LAP1. Blots were probed with Abs against LAP1, myosin heavy chain (MHC), and GAPDH. Lap1 shRNA #2 was selected as it gave the greatest LAP1 depletion. (**B**) IBs of primary hepatocyte lysates probed with anti-LAP1 and anti-**γ**-tubulin Abs. Hepatocytes isolated from a WT mouse were transduced with adenoviral vectors that expressed either control scrambled shRNA (Ad-ctrl) or shRNA that targets LAP1 (Ad-Lap1) in a dose of multiplicity of infection 50 at the time of plating and collected 48 h after transduction. (**C**) Relative ratios of band densities of LAP1A/B and LAP1C to γ-tubulin in IBs shown in panel B. Values are means ± SEM with each circle or square representing the value from each lane of the IB shown in panel B. ***P <0.001, ****P <0.0001 by Student's t-test. (**D**) Confocal immunofluorescence micrographs of hepatocytes isolated from a WT mouse administered the indicated adenovirus construct at a dose of 2 x 10^9 PFU/kg. Hepatocytes were labeled anti-LAP1 Ab (green) and DAPI (blue). Scale bars: 10 µm. (**E**) Immunofluorescence micrographs of liver sections stained anti-LAP1 Ab (green) and DAPI (blue). Five d post-injection of WT mice with Ad-ctrl or Ad-Lap1 shRNA, liver tissues were collected and fixed. Scale bars: 50 µm.

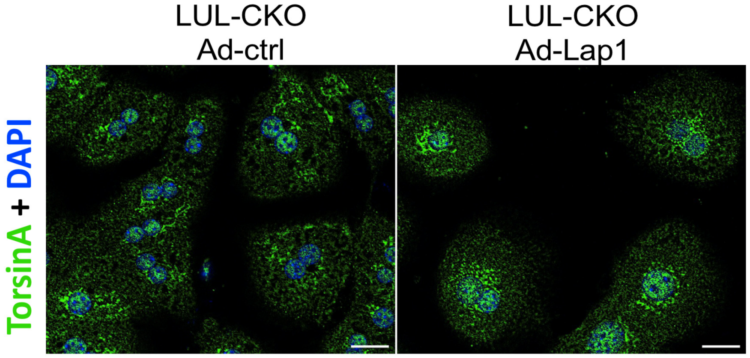

Supplemental Figure 8. Immunofluorescence confocal micrographs of torsinA distribution in hepatocytes with depletion of LULL1 or combined depletion LAP1 and LULL1. Immunofluorescence confocal micrographs of primary hepatocytes isolated from LUL-CKO mice injected with either Ad-ctrl (depleted of LULL1) or Ad-Lap1 (depleted of both LAP1 and LULL1). Cells were stained with anti-torsinA Ab (green) and DAPI (blue). Scale bars: 20 µm.

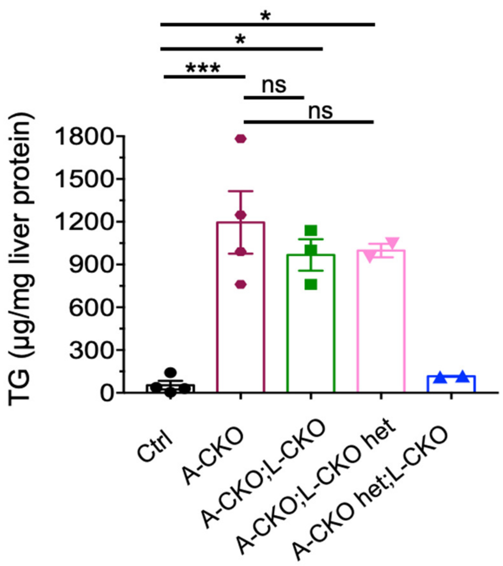

Supplemental Figure 9. Liver TG content in mice with combined depletion of LAP1 and torsinA from hepatocytes. Mice were fasted for 4-5 h before collecting livers to measure TG content. Values are means ± SEM with each symbol representing the value from an individual mouse of the indicated genotype (n = 2-4). *P < 0.05, ***P < 0.001, ns = not significant by ANOVA.

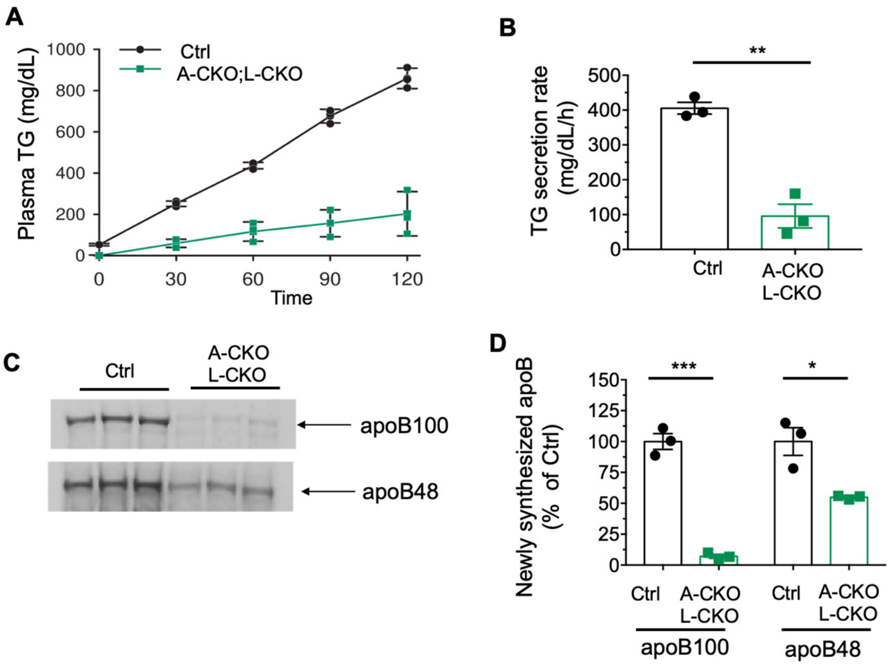

Supplemental Figure 10. TG secretion in mice with depletion of LAP1 and torsinA from hepatocytes. (**A**) Plasma TG concentrations versus time after injection of tyloxapol to block peripheral uptake in control (Ctrl) and A-CKO;L-CKO mice. Values are means ± SEM with each circle or square representing the value for an individual mouse (n = 3). (**B**) TG secretion rates were calculated by changes in plasma concentrations between the 30 and 120 min time points in a. Values are means ± SEM. Each circle or square indicates values from individual mouse. **$p < 0.01$ by Student's t-test. (**C**) Autoradiogram of SDS-polyacrylamide gel showing ^{35}S-labeled proteins collected 60 min after injections with ^{35}S-methionine. Each lane shows proteins from an individual mouse (n = 3 per group). Migrations of ^{35}S-labeled apoB100 and apoB48 are indicated. (**D**) Bands corresponding to apoB100 and apoB48 shown in c were quantified by densitometry and shown as a % of the value of control. Values are means ± SEM. Each circle or square indicates values from individual mouse (n = 3). *$p < 0.05$, ***$p < 0.001$ by Student's t-test.

Supplemental Table 1. Sequences of primers used for genotyping of LUL-flox and LUL-CKO mice

**Primer (see Supplemental Figure 1)**	**Sequence**
Lox gtF	acgggaaacaaaaggaggtt
Lox gtR	gatggctcagcggttaagag
Frt gtF	aatgggtgtgcggttgtta
Frt gtR	tggtcaagagagagccaggt

Supplemental Table 2. Quantification of mRNAs for LAP1B/C, LULL1 and HPRT in mouse liver*

Encoded Protein	Mean CT	SEM
LAP1B/C	22.83	0.07
LULL1	23.25	0.06
HPRT	25.63	0.08

*Total RNAs were used for quantitative real-time PCR. Mean cycle threshold (CT) values and SEM were calculated from values from 4 different mice per group. The primers for LAP1 detect mRNAs for isoforms LAP1B and LAP1C. The sequences of primers used are provided in the Supplemental Table 3.

Supplemental Table 3. Primers used for quantitative real-time PCR to detect mRNAs encoding LAP1B/C, LULL1 and HPRT

**Protein**	**Sequence**
LAP1B/C forward primer	tgcagacccccattaagaag
LAP1B/C reverse primer	ggatctggccctgatagtga
LULL1 forward primer	cagcagtggttccctagtttag
LULL1 reverse primer	ggcagtagtcagccctgttaat
HPRT forward primer	agttgagagatcatctccac
HPRT reverse primer	ttgctgacctgctggatttac
