# Supplementary material for: Dynamic regulation of hepatic lipid metabolism by torsinA and its activators
Source: JCI Insight. 2024 Jan 9;9(3):e175328. doi: 10.1172/jci.insight.175328 (PMC10967386; doi:10.1172/jci.insight.175328)

Full unedited gel for Figure 1C

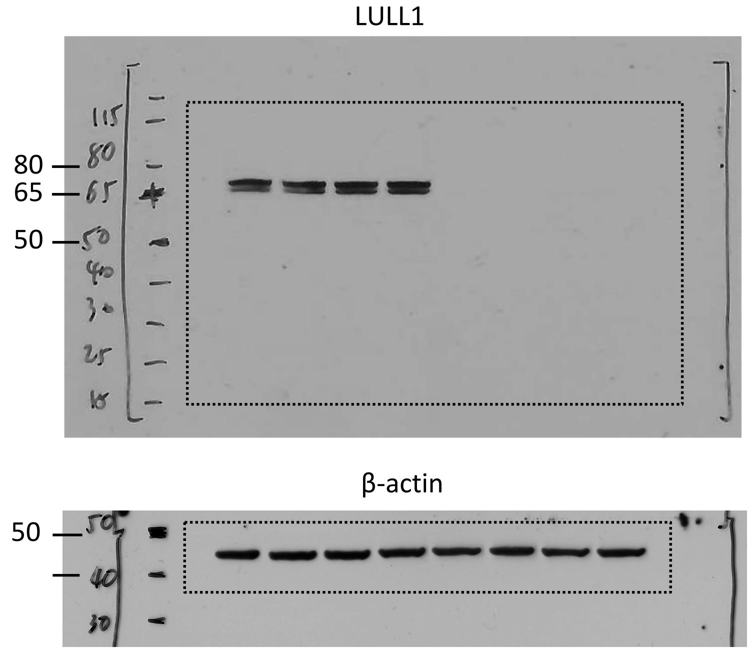

Full unedited gel Figure 1H

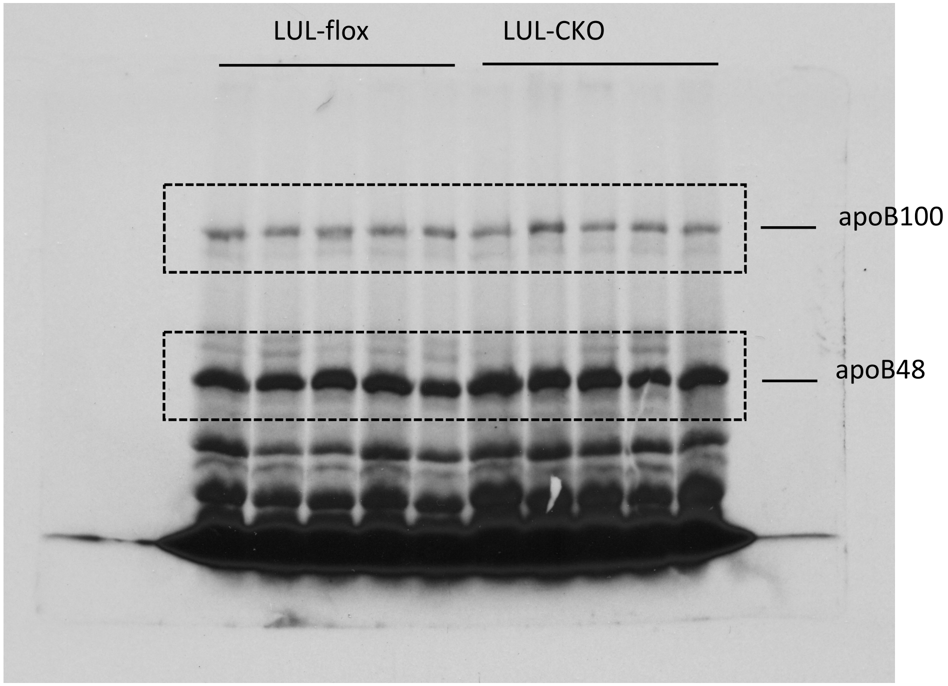

Full unedited gel for Figure 2A

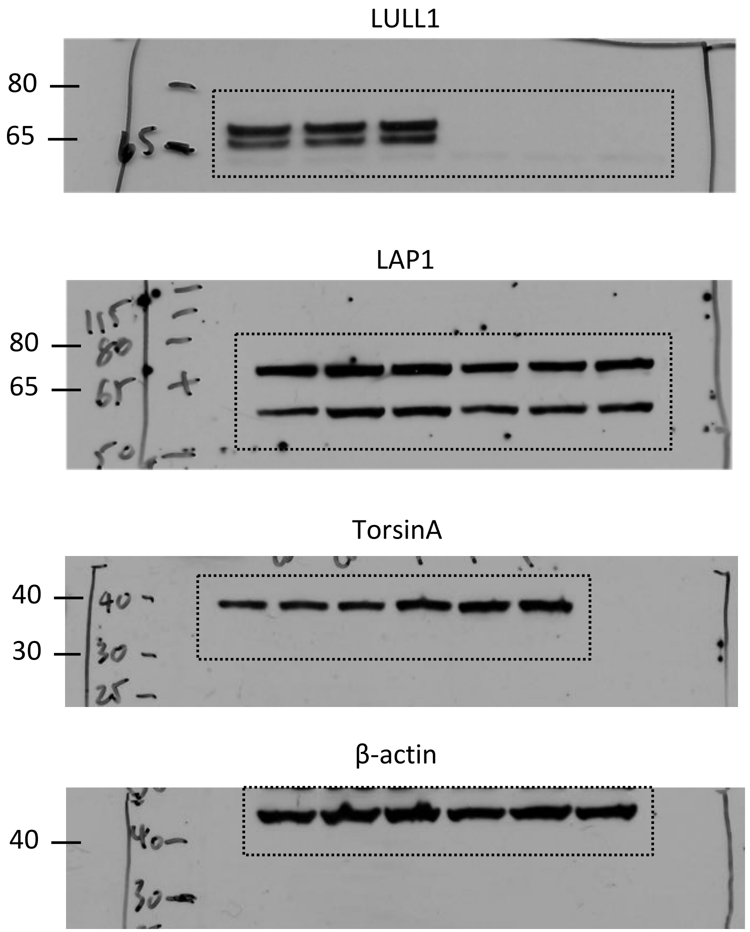

Full unedited gel for Figure 3A

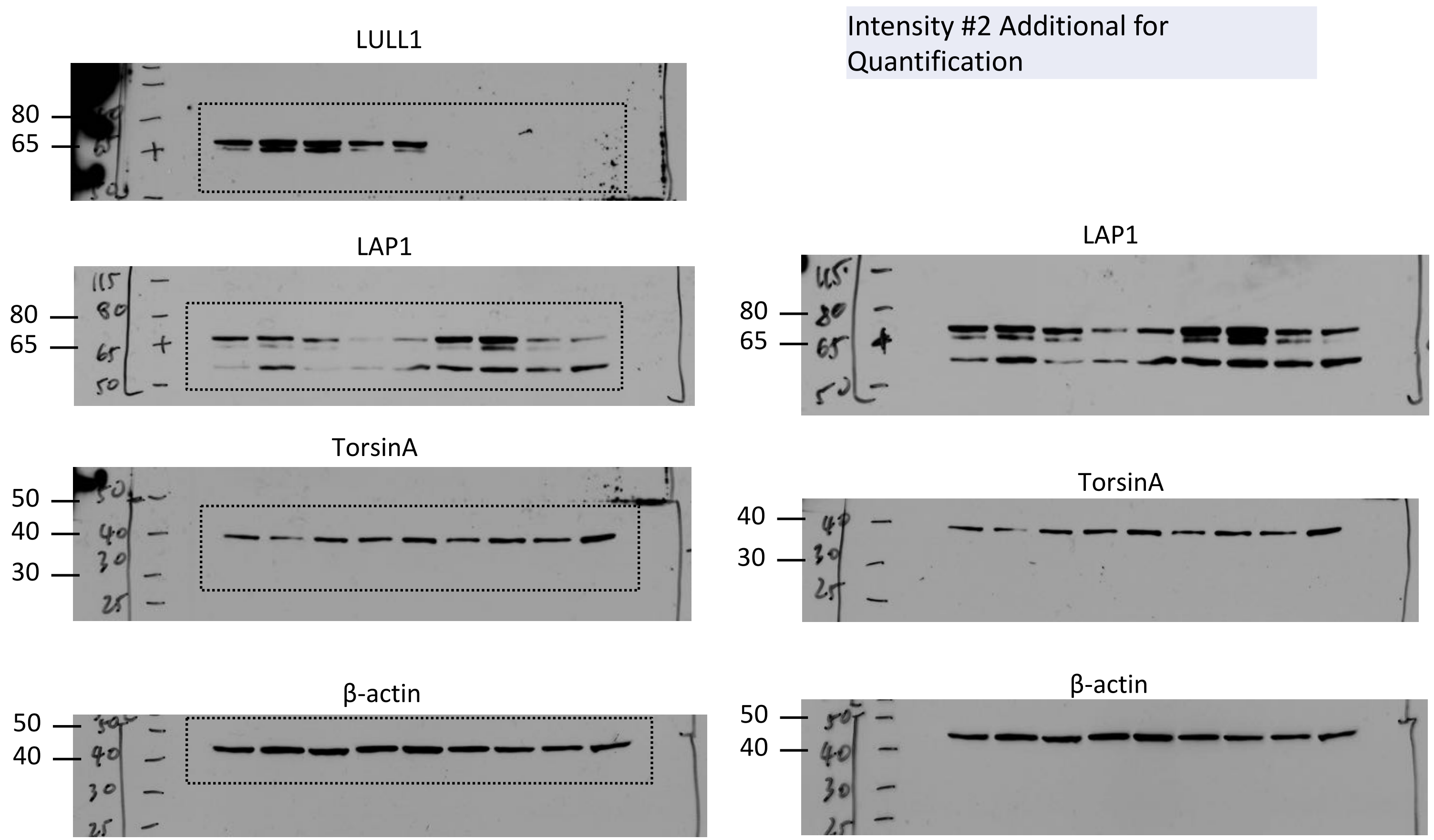

Full unedited gel for Figure 4A

LULL1

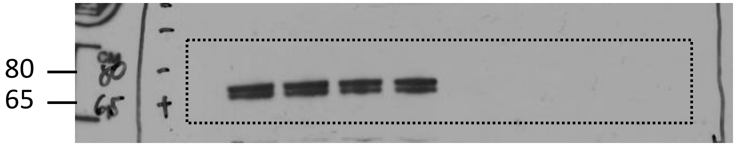

LAP1

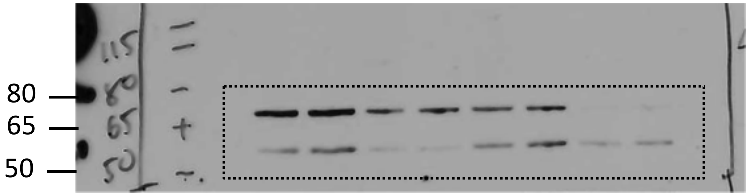

TorsinA

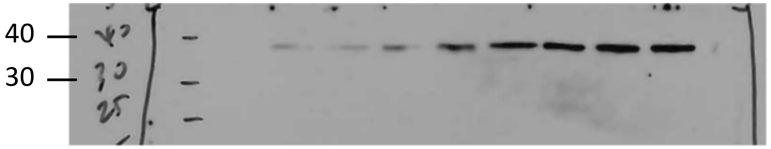

β-actin

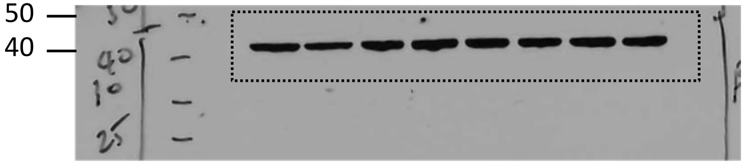

Intensity #2 Additional for Quantification

LAP1

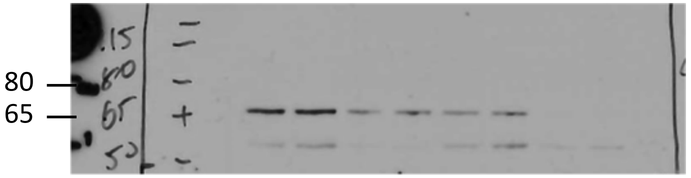

TorsinA

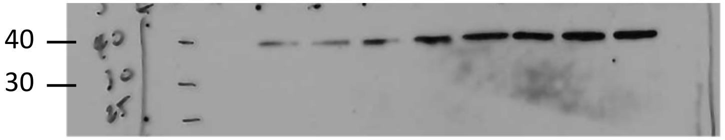

β-actin

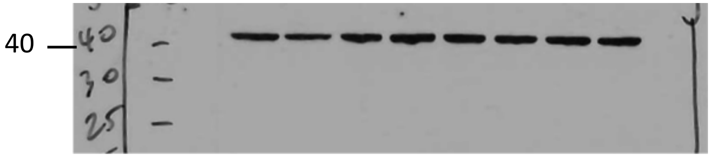

Full unedited gel for Figure 6C

Experiment date: 9/28/21

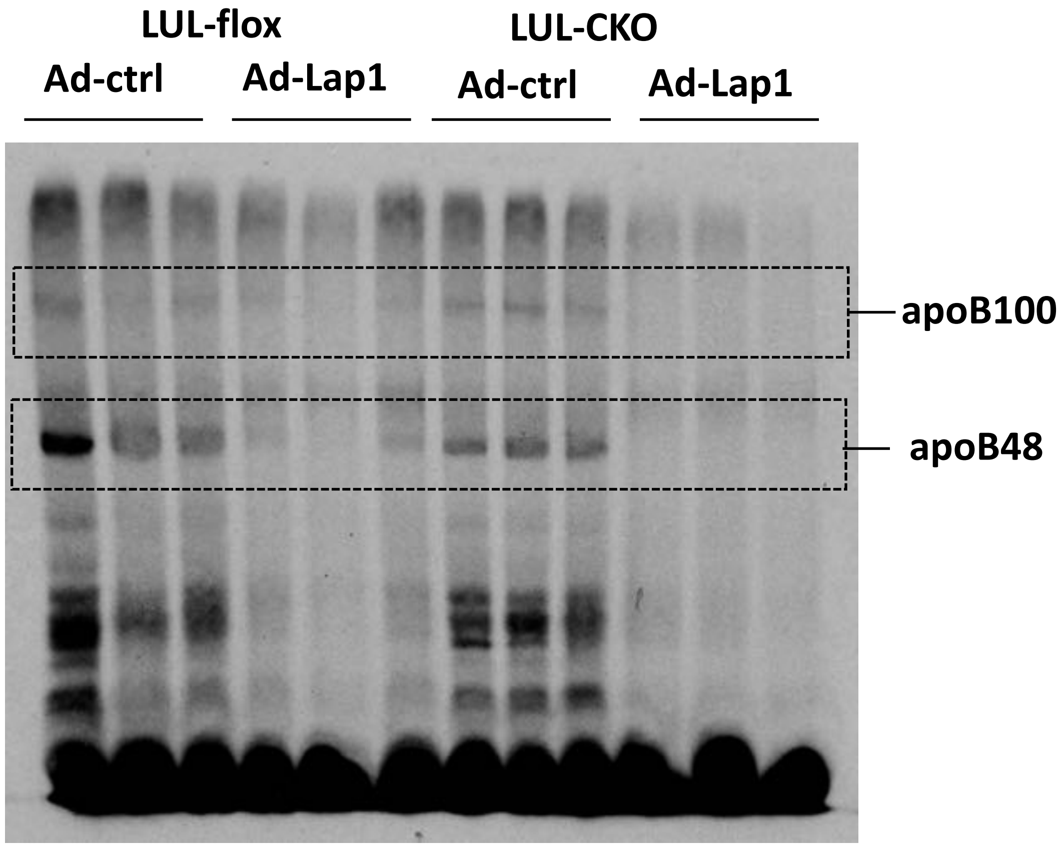

Experiment date: 8/2/23

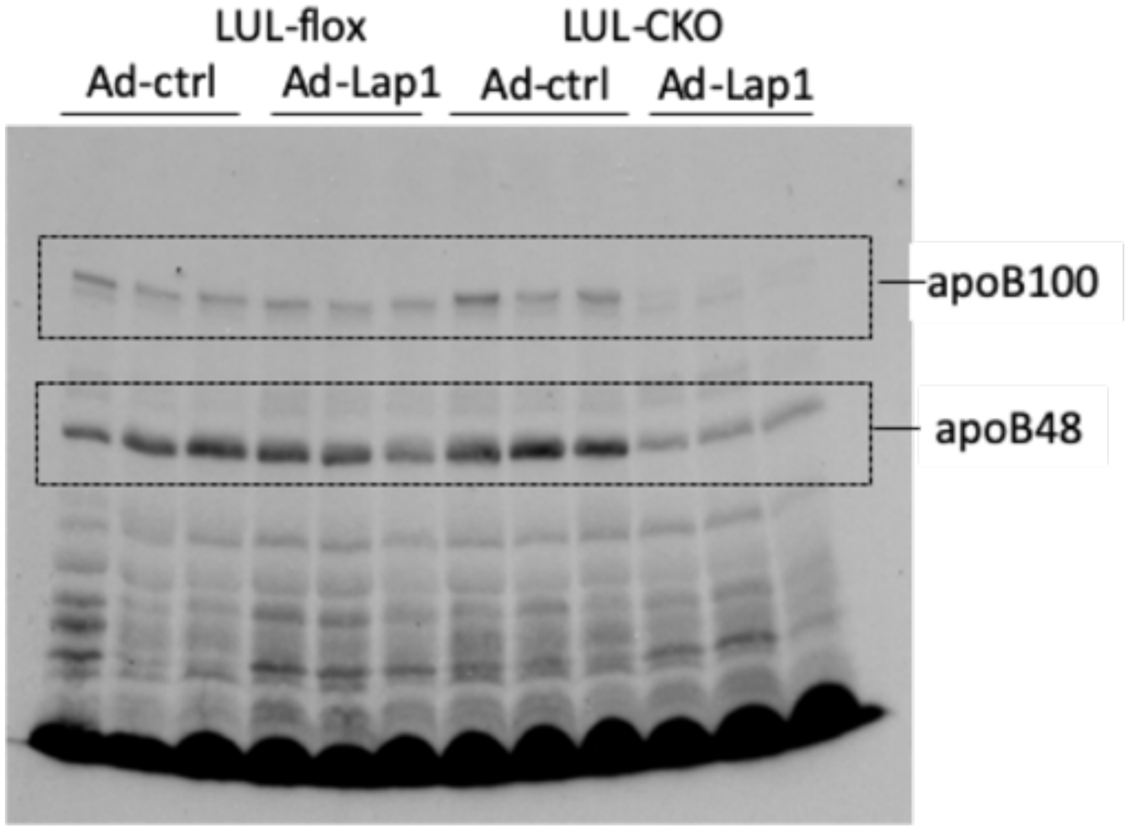

Full unedited gel for Supplemental Figure 2

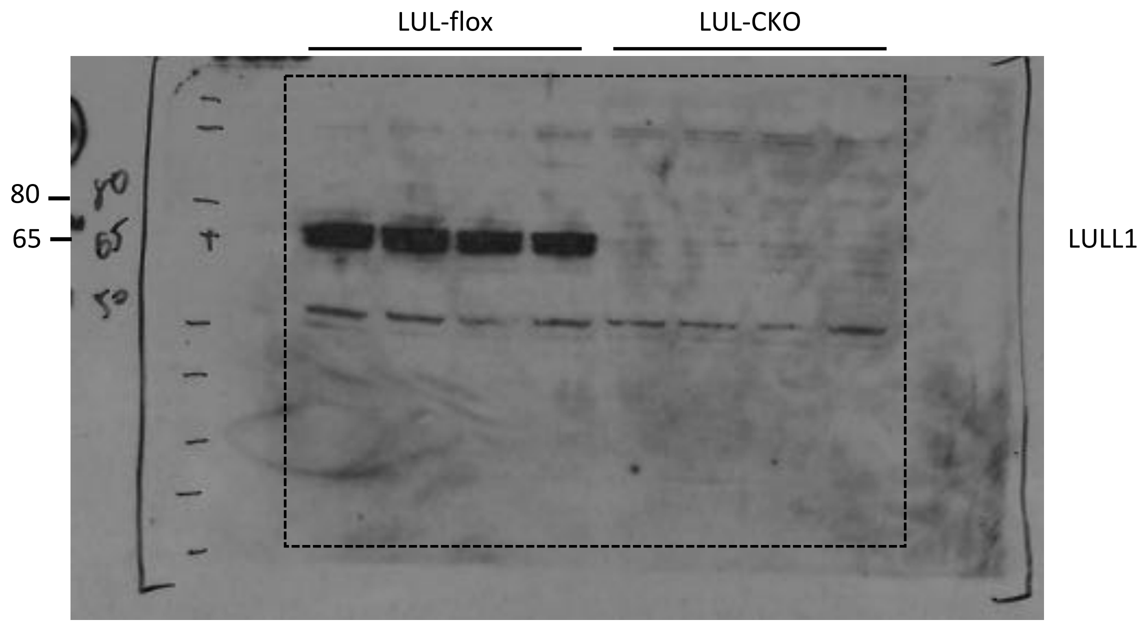

Full unedited gel for Supplemental Figure 4A

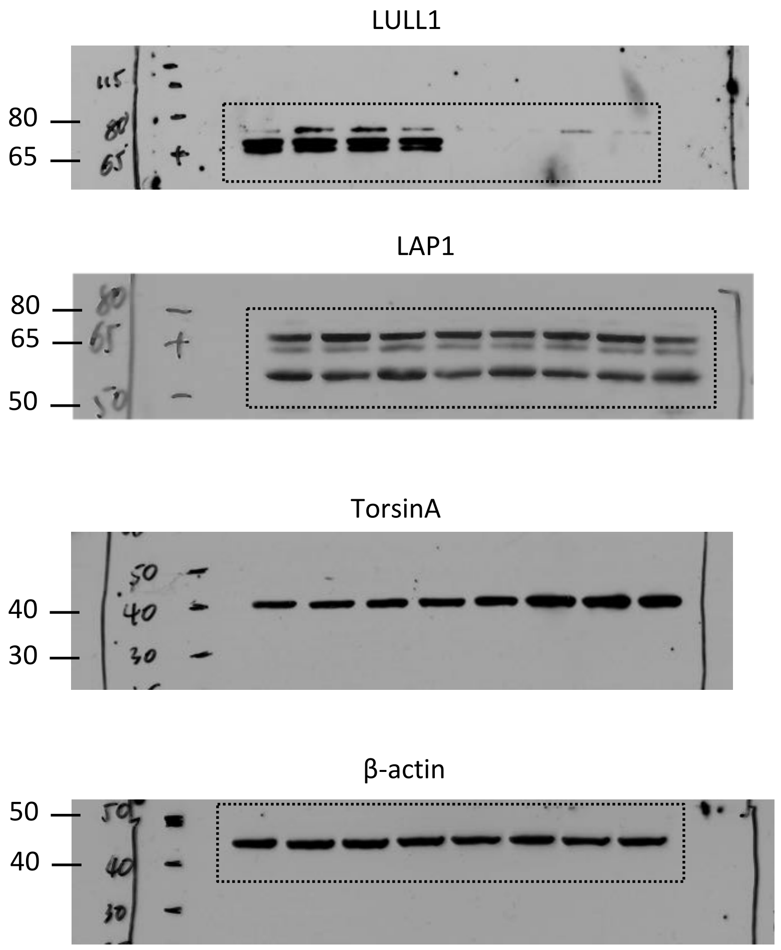

Full unedited gel for Supplemental Figure 6

LAP1

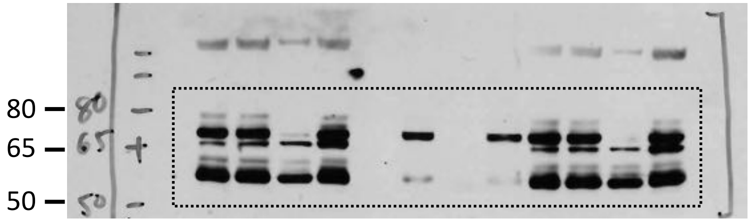

LULL1

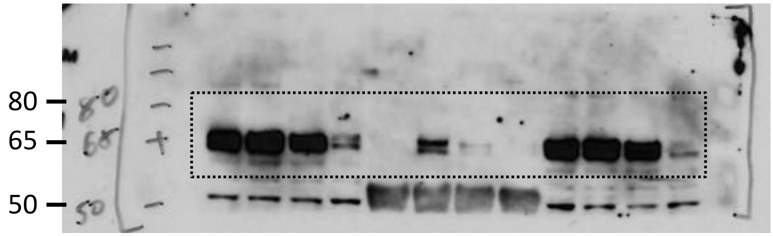

TorsinA

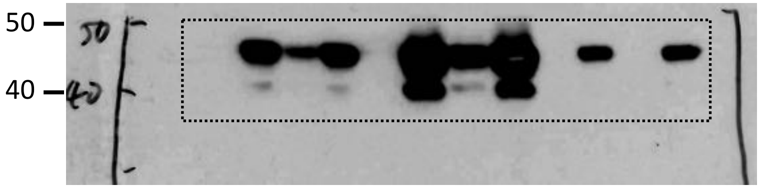

Flag

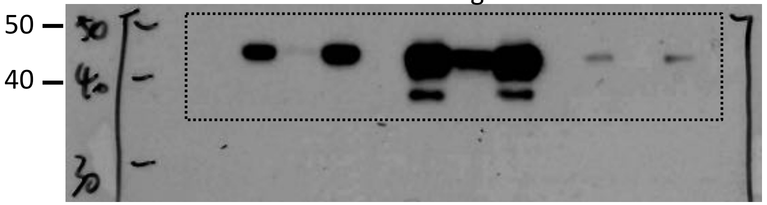

Full unedited gel for Supplemental Figure 7A

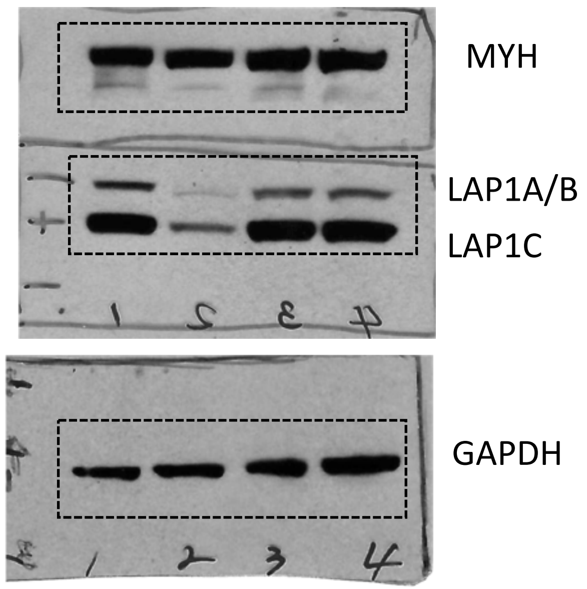

Full unedited gel for Supplemental Figure 7B

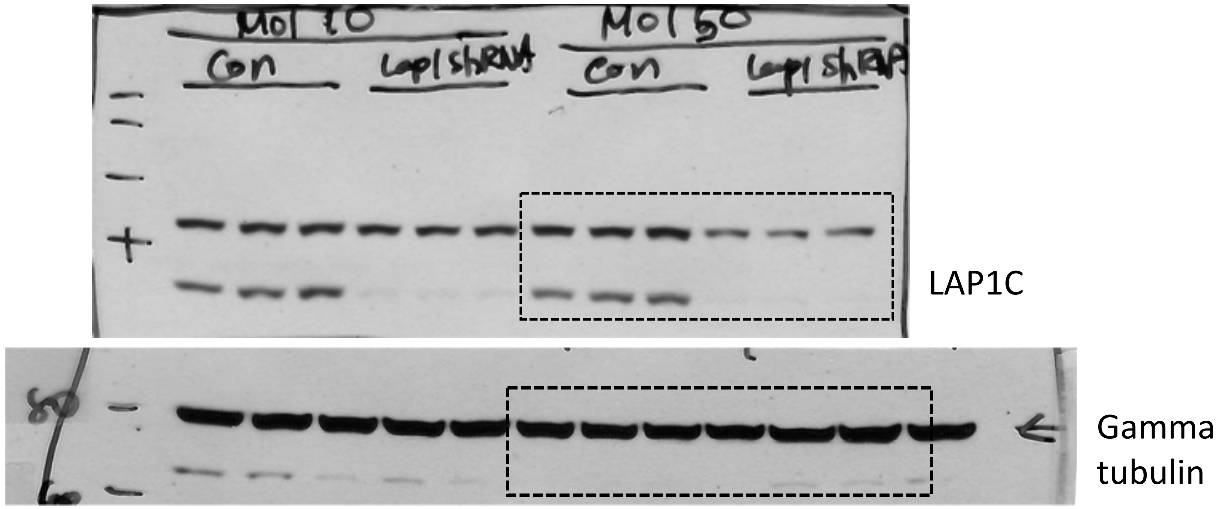

Full unedited gel for Supplemental Figure 10C

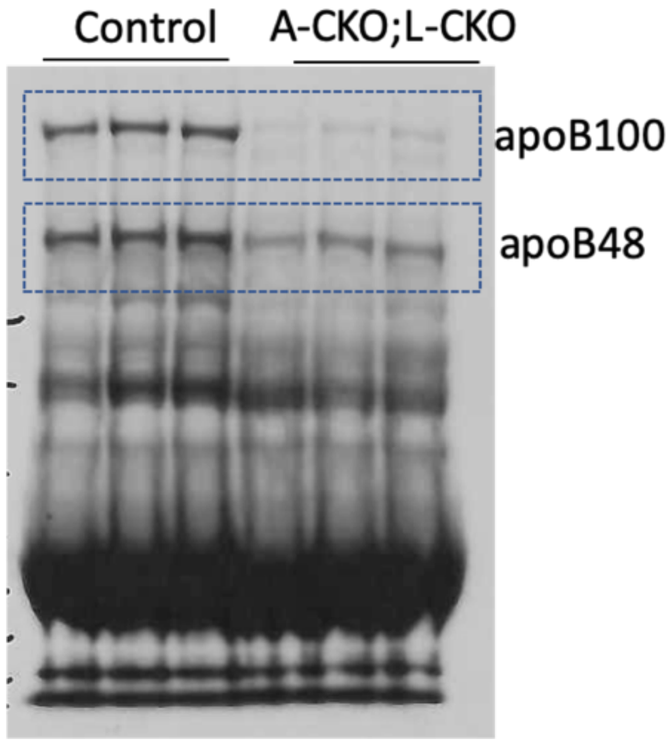

Supplement: Unedited blot and gel images [file jciinsight-9-175328-s009.pdf]
